# Diagnostic Biomarkers for Risk Estimation of In-Hospital and Post-Discharge Cardiovascular Mortality in ST-Segment Elevation Myocardial Infarction (STEMI) Patients

**DOI:** 10.3390/jcm14186632

**Published:** 2025-09-20

**Authors:** Kristen Kopp, Michael Lichtenauer, Vera Paar, Uta C. Hoppe, Rozana F. Rakhimova, Elena A. Badykova, Eduard F. Agletdinov, Dimitry M. Grishaev, Ksenia A. Cheremisina, Anastasia V. Baraboshkina, Irina A. Lakman, Liya R. Abzalilova, Naufal S. Zagidullin

**Affiliations:** 1 Department of Internal Medicine II, Division of Cardiology, Paracelsus Medical University, 5020 Salzburg, Austria; m.lichtenauer@salk.at (M.L.); v.paar@salk.at (V.P.); u.hoppe@salk.at (U.C.H.); 2 Department of Internal Medicine, Bashkir State Medical University, 450008 Ufa, Russia; r.r-7@mail.ru (R.F.R.); lnurova@mail.ru (E.A.B.); 3 Vector-Best RIDT JSC, 630117 Novosibirsk, Russia; agletdinov@vector-best.ru (E.F.A.); grishaevd@vector-best.ru (D.M.G.); cheremisina@vector-best.ru (K.A.C.); baraboshkina@vector-best.ru (A.V.B.); 4 Department of Statistics and Business Informatics, Ufa University of Science and Technology, 450076 Ufa, Russia; lackmania@mail.ru (I.A.L.); abzalilova.liya@gmail.com (L.R.A.)

**Keywords:** STEMI, biomarkers, CRP, sST2, H-FABP, cardiovascular mortality, risk prediction

## Abstract

**Background**: ST-segment-elevation myocardial infarction (STEMI) continues to be associated with substantial short- and long-term cardiovascular (CV) mortality despite advances in treatment. Accurate early risk stratification remains critical for optimizing outcomes. Emerging biomarkers including CRP, sST2, and FABP may enhance predictive precision beyond classical markers. This study aimed to evaluate the prognostic value of these biomarkers for in-hospital and 18-month post-discharge CV mortality in STEMI patients. **Methods**: In this prospective, single-center study, 179 consecutive STEMI patients admitted September 2020–June 2021 underwent biomarker evaluation upon admission. Serum concentrations of CRP, sST2, and H-FABP were measured by ELISA. Patients were followed for in-hospital outcomes and post-discharge mortality during 18-month follow-up (FU) (last patient, last visit January 2023). ROC analysis was used to determine biomarker cut-off values. Cox regression and Kaplan-Meier analyses assessed associations with mortality. **Results**: In-hospital mortality was 7.8% (14/179). Elevated CRP (>11 mg/L) was significantly associated with higher in-hospital mortality (21.4% vs. 3.7%, *p* < 0.01). sST2 and H-FABP showed trends toward worse outcomes at higher levels, although their independent predictive value was less robust. Cox regression identified CRP > 11 mg/L (HR = 4.93, *p* < 0.01), admission glucose, and reduced GFR as independent predictors of in-hospital mortality. During FU, 18 of 165 discharged patients (10.1%) experienced CV death. Higher sST2 levels were significantly associated with post-discharge mortality in midterm FU (*p* = 0.041). **Conclusions**: We could show that CRP > 11 mg/L is a strong predictor of in-hospital mortality while elevated sST2 is associated with CV mortality during midterm FU in STEMI patients. Incorporating these biomarkers into clinical risk models may enhance early risk prediction and identify patients at higher risk for post-discharge events.

## 1. Introduction

Despite the development of new therapeutic strategies, coronary artery disease (CAD) remains one of the main health burdens worldwide. The occurrence of ST-segment elevation myocardial infarction (STEMI) is especially associated with significant short- and long-term complications. STEMI patients are at higher risk of cardiovascular (CV) events, even in a long-term post-myocardial infarction (MI) period, resulting in consequent reduction of long-term survival in this population [1].

Therefore, early identification of high-risk individuals is one of the main goals in daily clinical practice in these patients. The use of biological markers has been shown to improve the accuracy of diagnosis in CV patients. Indeed, this approach promotes stratification of CV risk, both during the hospitalization period as well as in the long-term observation period. Levels of several biomarkers correlate with the severity of CV disease, reflect the dynamics of disease and enhance the efficacy of therapy regimes. “Classic” biomarkers like myoglobin fraction of creatine phosphokinase (CK-MB) and troponins correlate with the long-term outcome of STEMI patients and are integrated into daily clinical practice [1] However, additional tools are needed to promote the estimation of CV outcome for the individual patient.

Multimarker analytic approaches have been shown to enhance the sensitivity and specificity of prognostic assessments. Consequently, they might be a more effective tool in predicting CV mortality in MI patients in the acute and follow-up (FU) period. Serum biomarkers like ST2 (Suppression of Tumorigenicity 2 protein), FABP (fatty acid binding protein), and CRP (C-reactive protein) have emerged as a potentially useful tool for improving the assessment of CV disease. Soluble ST2 (sST2) is a member of the interleukin 1 (IL-1) receptor family. Its role in cardiac pathophysiological processes including the progression of coronary atherosclerosis but also other cardiac remodeling processes was established in recent years [2]. Indeed, sST2 seems to not only participate in CV response to injury but also in myocardial remodeling processes observed in heart failure (HF) and MI [3,4]. Serum concentrations of sST2 correlate with the outcome of MI and also HF patients [5,6] and even patients with COVID-19 [7]. The fatty-acid-binding proteins (FABPs) are a family of transport proteins for fatty acids and other lipophilic substances such as eicosanoids and retinoids. Heart-type FABP (H-FABP) is a low molecular weight cytosolic protein, which is primarily expressed in myocardial tissue and functions as the principal transporter of long-chain fatty acids in the cardiomyocyte [8]. Under normal conditions, H-FABP is released into the blood upon cardiac cellular injury within 2 h (h), peaks at about 4–6 h [9], and thus could be used as an early marker of acute MI due to its high sensitivity, specificity and prognostic value. CRP circulates the serum, however, in some conditions, including acute inflammatory processes, tissue injuries, or acute infections, its marked increase in circulation can be observed. Some cardiac conditions, such as coronary heart disease (CHD), ventricular hypertrophy, HF, and even valvular heart diseases have underlying inflammatory etiologies accompanied by increased levels of inflammatory responses and thus CRP may have a major role to predict various types of CV diseases even in healthy subjects [10,11,12]. Nevertheless, the ability of the aforementioned biomarkers to assess outcomes in MI patients still remains a matter of debate. Importantly, the question of whether the biomarkers CRP, H-FABP and sST2, can improve predictive sensitivity as well as specificity of in-hospital CV mortality risk as well as risk of CV mortality during FU in STEMI patients has not been exhaustively explored.

The aim of the study was to investigate serum levels of CRP, H-FABP, and sST2 in STEMI patients to predict in-hospital mortality and CV mortality during 18-month midterm FU.

## 2. Materials and Methods

In this prospective, non-randomized, single-center study, 179 consecutive patients were hospitalized and treated for acute STEMI at the Department of Cardiology at the Ufa City Hospital N21, Russian Federation offering 24/7 percutaneous coronary intervention (PCI) services. Patients were enrolled in the study between September 2020 and June 2021 and followed for 18 months (last patient, last follow-up January 2023). Initial diagnosis was established by 12-lead electrocardiogram (ECG) upon admission according to European Guidelines criteria [13]. ST-segment elevation was measured at the J-point at least in two contiguous leads with ST-segment elevation of 2.5 mm (mm) in men < 40 years, 2 mm in men 40 years, or 1.5 mm in women in leads V2–V3 and/or 1 mm in the other leads in the absence of left ventricular hypertrophy or left bundle branch block. The diagnosis was verified during clinical FU by further ECG recordings (day two and/or day three of hospital stay), transthoracic echocardiographic (day two or day three of hospital stay), laboratory (hs-Troponin I and CK-MB at admission and during FU at day two and/or day three of hospital stay) and coronary angiography (CAG) according to the 2023 European Society of Cardiology (ESC) guidelines [1].

Dependent on the time of STEMI diagnosis, acute CAG/PCI was performed if patient presentation to the cardiac catheterization laboratory was ≤120 min (min) or acute thrombolysis was given presentation by ≤12 h time from symptom presentation and no contraindication for thrombolytic therapy as established by and emergency doctor or primary care physician. If patients presented with signs of failed fibrinolysis, or by evidence of re-occlusion or re-infarction with ST-segment dynamics, rescue PCI was performed as soon as possible. Acute medical treatment, including an antiplatelet regimen and post-MI guideline-recommended medications were given according to the ESC guidelines (Table 1) during baseline hospitalization and at discharge [1]. Capture of further relevant diagnoses was obtained according to medical history, clinical findings, ECG, laboratory data and transthoracic echocardiography reports.

The study was approved by the Ethics Committee of Bashkir State Medical University (N1 from 23 January 2017) and was performed in accordance with standards of good clinical practice (GCP) and the principles of the Declaration of Helsinki. Prior to inclusion, all participants signed an informed consent.

The inclusion criteria were: age > 18 years and diagnosis of STEMI according to current ESC guidelines (see above). The exclusion criteria were: >48 h from start of typical symptoms of acute coronary syndrome (ACS), severe valvular dysfunction defined as severe regurgitation or stenosis of one or more of the cardiac valves, dilative cardiomyopathy, permanent atrial fibrillation (AF) and/or atrial flutter, AV block II–III according to medical history and ECG, implanted pacemaker, acute pulmonary embolism, active malignant disease defined as achieved tumor-free survival under three years, severe chronic obstructive pulmonary disease (GOLD 2009 stage III–IV), uncontrolled bronchial asthma (according to Global Initiative for Asthma, GINA 2019), acute infectious diseases at the time of STEMI defined as acute pyelonephritis, community-acquired pneumonia, acute bronchitis and/or flu/acute respiratory viral infection, and kidney failure defined as glomerular filtration rate (GFR) < 30 mL/min 1.73 m^2^, as well as pregnancy or lactation.

**Figure 1:**
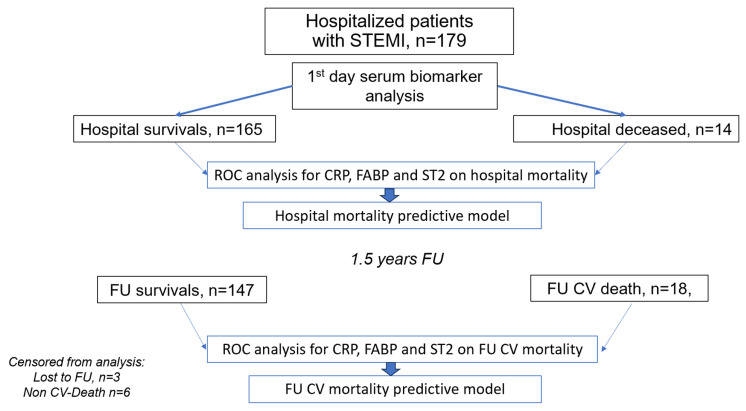
Study design.

Patient enrollment and the design of the study are presented in Figure 1. On the day of hospital admission, patients’ venous blood was drawn for the study prior to PCI (≤8 h), subsequently centrifuged and the serum was frozen for further analyses. Blood serum samples were anonymized, aliquoted, and frozen immediately at −70 °C.

The serum concentrations of biomarkers CRP, H-FABP, and sST2 were analyzed by enzyme immunoassay (ELISA) as indicated by the manufacturer “Vector-Best”, Russia using calibrated, certified, and maintained equipment with technicians blinded to study outcomes.

CRP was measured using the kit with the catalog number (cat. №A-9002 CRP-EIA-BEST). The analytical range of measurements was 0–10 mg/L. The coefficient of variation within a batch (intra-assay CV) was <8%, respectively. Lot number (lot): 115.

H-FABP was measured using the kit with the catalog number A-9104-H-FABP EIA-BEST. The analytical range of measurements was 0–15 ng/mL. The coefficient of variation within a batch (intra-assay CV) was <8%, respectively. Lot number (lot): 48.

sST2 was measured using the kit with the catalog number A-9110-sST2 EIA-BEST. The analytical range of measurements was 0–140 ng/mL. The coefficient of variation within a batch (intra-assay CV) was <8%, respectively. Batch number (lot): 12.

Samples were thawed only once. Storage was for no more than 6 months before analysis according to the manufacturer’s specification. All samples were analyzed in a single set of experiments to minimize variability. The inter-batch variation coefficient is not provided, since all samples were analyzed in one batch.

A detailed medical history was obtained at admission for all enrolled patients, including current clinical symptoms, as well as history of previous illnesses, and current medications.

All eligible patients were examined during baseline hospitalization and followed for 18 months to capture CV mortality. Survival status at 18 months (552 ± 42 days) was recorded using a remote data capture system “ProMed” (Program for Medical Cases Monitoring).

Demographic variables captured in the analysis included age, sex, Body Mass Index (BMI), concomitant CV disease, CHD, arterial hypertension (AH), diabetes mellitus (DM), chronic kidney disease (CKD), prior MI, acute/chronic HF, prior stroke, chronic obstructive pulmonary disease (COPD). The primary endpoints were in-hospital mortality and CV mortality at 18 months post-discharge.

Statistical analysis was carried out by our blinded statistical analytic team using SPSS software package 21 (IBM SPSS Statistics for Windows, Version 21.0. Armonk, NY, USA: IBM Corp.) and R Studio version 4.4.3 (Posit team (2025). RStudio: Integrated Development Environment for R. Posit Software, PBC, Boston, MA, USA).

Preliminary Shapiro-Wilk testing showed non-normal distribution, therefore median and interquartile range (Q1; Q3) were used to describe the distribution of continuous variables. For the description of categorical variables, absolute and relative frequencies were used. The Mann-Whitney test was applied to determine the statistical significance of difference between medians of continuous variables, while Chi-square testing was applied for analysis of categorical variables and Yates correction was applied if necessary.

Kaplan-Meier multiple estimator methods were used to compare in-hospital survival between groups, and the Gahan-Wilcoxon test was used to evaluate survival outcomes defined significant at *p* < 0.05. The target variable defining the duration of the event state is given in days for in-hospital survival and in months for FU survival. Univariate Cox proportional hazards models for binarized by cut-off points of CRP, H-FABP, and sST2 biomarkers were estimated to identify predictors of the risk of in-hospital death. Model estimates were controlled using the likelihood ratio test (LR test) with the null hypothesis of the model as a whole and the difference from one of the values of the Harrell confidence interval.

Alternatively, Cox models with restricted cubic splines (RCS) were also built, where transformed values of baseline CRP, H-FABP, and sST2 levels were used as predictors. For right-skewed H-FABP and sST2, logarithm was used as a transformation, for left-skewed CRP levels, their values were inverted before re-characterization (baseline CRP levels were subtracted from the maximum value +1). The number of nodes for splines was selected by enumeration from 3 to 5, based on the minimum values of the Akaike information criterion. To visualize the results of Cox with RCS modeling, a graph of the dependence of HR on the baseline values of biomarker levels was constructed. For modeling hospital survival, the following R libraries were used: “rms”, “survival”, “ggplot2”, “survminer”.

Since we had competing events (“Alive”, “Cardiovascular deaths”, “Non-cardiovascular deaths”) in the FU 18-month observation, their cumulative probability was calculated using Fine-Gray estimates for competing risks of death. The Fine test was also performed to assess the significance of the effect of biomarker levels exceeding their cutoff points on the risk of death (here the parameter of interest is cardiovascular deaths). Event-free survival (EFS) was also assessed. To assess the risk of death from cardiovascular causes, two-factor Fine-Gray models with competing risks were used, where biomarker levels (baseline, transformed, and binarized) and patient age were considered as factors. As with in-hospital survival, logarithmic transformation was performed as a transformation for right-skewed sST2 and H-FABP, and for left-skewed CRP, the values were “inverted” from higher to lower before logarithmic transformation. The interpretation of the results is based on the calculation of the hazard ratio (HR) and confidence interval with 95% reliability for each reliable risk predictor. The following R libraries were used to model post-hospital survival: “tidycmprsk”, “survival”, “ggsurvfit”.

## 3. Results

### 3.1. In-Hospital Mortality

Table 1 presents the characteristics (column “all”) of the study population as well as the in-hospital treatment and discharge therapy regime. In summary, the majority of study patients were men (n = 124) compared to women (n = 55). Patients presented with typical comorbidities observed in the CAD population, including arterial hypertension, dyslipidemia and DM. If manageable, in-hospital treatment and discharge regimen adhered to current ESC guidelines, as indicated above [1]. 175 patients underwent primary PCI with a success rate of 97.1% (170/175), and 10 (5.6%) patients were treated by acute thrombolytic therapy. In most patients, the left coronary artery (LCA) was occluded (68.16%). In four patients, CAG was contraindicated or the patient refused. The success rate of the thrombolytic regime was 60.0% (6/10). 14 (7.8%) of 179 patients died and the characteristics by in-hospital survival or deceased status, respectively, are presented in Table 1. The groups were comparable in age, men/women ratio and BMI (*p* > 0.05). They differed in systolic and diastolic blood pressure (lower in deceased, *p* = 0.01 and *p* = 0.001, consequently), DM (*p* < 0.001), CKD (*p* = 0.005, both more in deceased) and successful stenting (less in deceased, *p* = 0.026). Deceased patients had higher pulmonary artery (PA) pressure (*p* = 0.022). The biochemical and ELISA tests are presented in Table 2 and the deceased group showed higher levels of glucose, N-terminal pro-B-type natriuretic peptide (NT-proBNP), cystatin C, Apo A1, CRP, sST2, creatinine and lactate dehydrogenase (LDH, *p* < 0.05), and lower—in GFR and LDL-C (*p* < 0.05).

#### 3.1.1. CRP and In-Hospital Mortality

**Figure 2:**
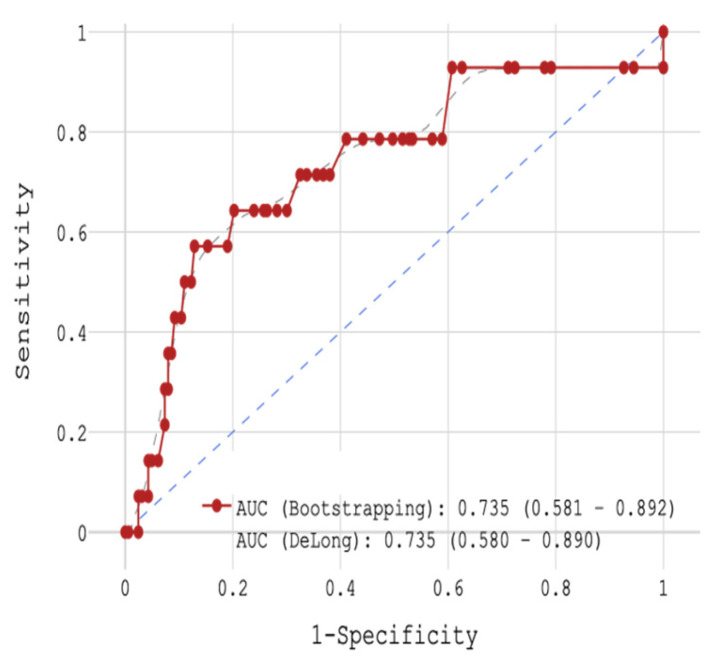
ROC-analysis of hospital mortality with STEMI patients depending on CRP concentration.

In the next stage, patients were analyzed regarding mortality with respect to CRP concentration. With the help of ROC analysis, the whole cohort was divided into 2 groups: (1) < the cut-off value of 11 mg/L (n = 135) or (2) ≥ the cut-off value of 11 mg/L (n = 42). The sensitivity was 64.3%, the specificity 79.8%, and the accuracy 78.5% (area under the curve (AUC) 0.735, Figure 2). In the 1st group, 3.7% of patients died in hospital (n = 5) and in the 2nd group, 9 patients died (21.4%) during their stay.

In Table 3 and Table 4, clinical and demographical characteristics of the patients in the groups with (1) < or (2) ≥ CRP cut-off value (11 mg/L) are presented. Patients in group 2 with CRP ≥ 11 mg/L had higher triglycerides levels (*p* < 0.001) and lower Apo A1 (*p* = 0.003).

**Figure 3:**
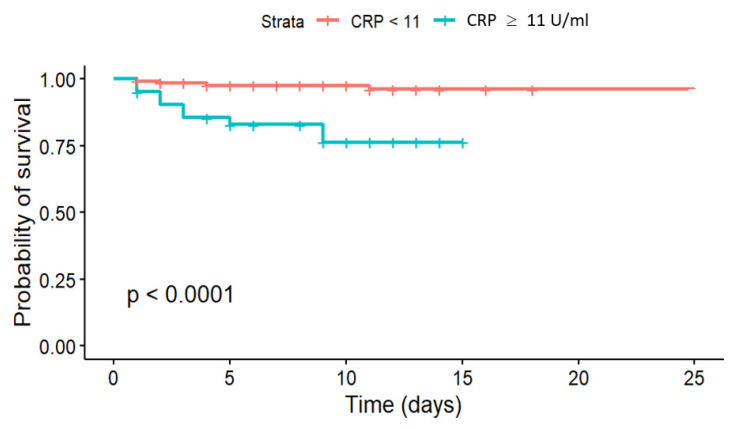
Kaplan-Meier hospital survival curves below and under CRP 11 mg/L cut-off point.

The results based on multiple Kaplan-Meier estimation analysis indicate the presence of significant differences between survival during the stay in hospital for patients with CRP > and ≤11 mg/L (Figure 3). The Gehan-Wilcoxon test confirmed the difference at *p* < 0.001.

#### 3.1.2. H-FABP and In-Hospital Mortality

**Figure 4:**
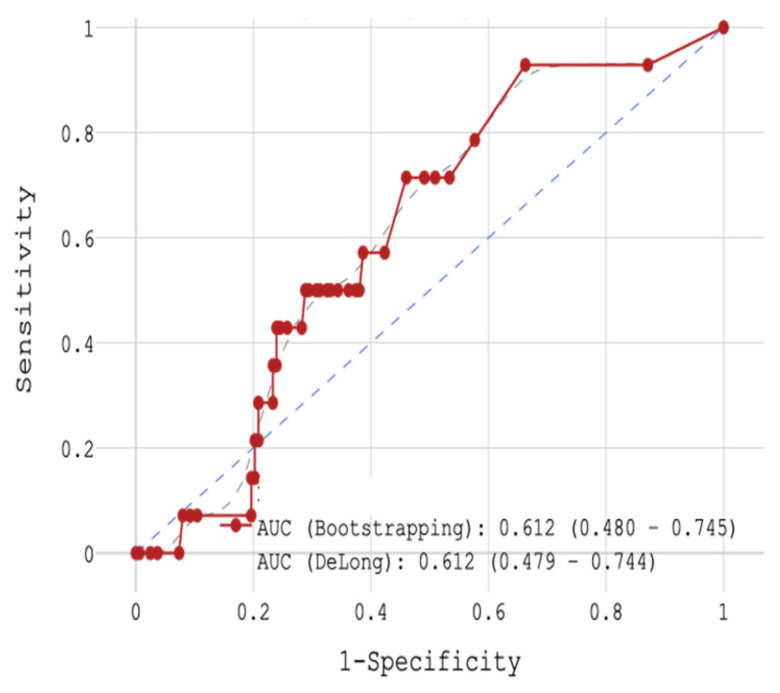
ROC-analysis by H-FABP in hospital mortality.

Next, in-hospital mortality depending on H-FABP concentration was analyzed. With the help of ROC-analysis the whole group was divided into 2 groups with (1) cut-off < 0.7 ng/mL (n = 92) and (2) cut-off > 0.7 ng/mL (n = 85). The sensitivity was 71.4%, specificity 54.1% and the accuracy 55.4% (AUC 0.612, Figure 4). The 1st group with H-FABP < 0.7 ng/mL had 4.35% mortality (n = 4), while the 2nd group ≥ 0.7 ng/mL had 11.76% mortality (n = 10).

In Table 5 and Table 6, a comparison between lower and higher H-FABP cut-off values is presented. Patients with higher H-FABP had lower systolic blood pressure (SBP) and diastolic blood pressure (DBP), and less frequent CKD (*p* = 0.040). In biochemical/ELISA analysis, CK-MB, TnI, Myoglobin and sST2 were significantly elevated in the higher H-FABP group (*p* < 0.05).

**Figure 5:**
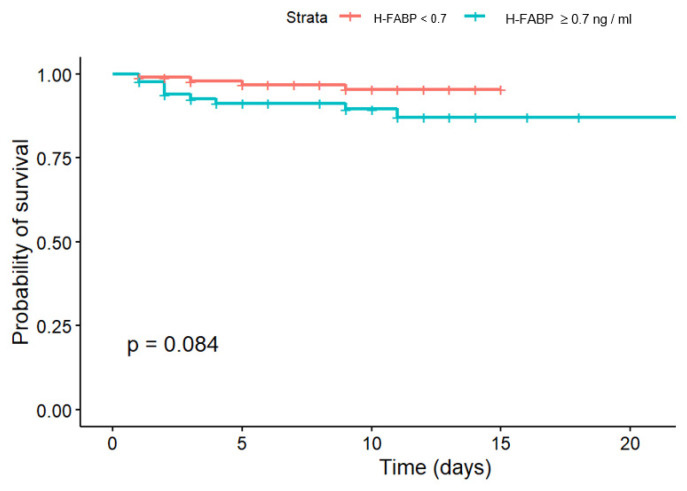
Kaplan-Meier hospital survival curves below and under H-FABP cut-off > 0.7 ng/mL.

Kaplan-Meier multiplier estimates determined for hospital survival in groups with H-FABP higher and lower than 0.7 ng/mL showed significant differences only at the *p* < 0.1 level (Figure 5). Gehan-Wilcoxon test confirmed the results (*p* = 0.084).

#### 3.1.3. sST2 and In-Hospital Mortality

**Figure 6:**
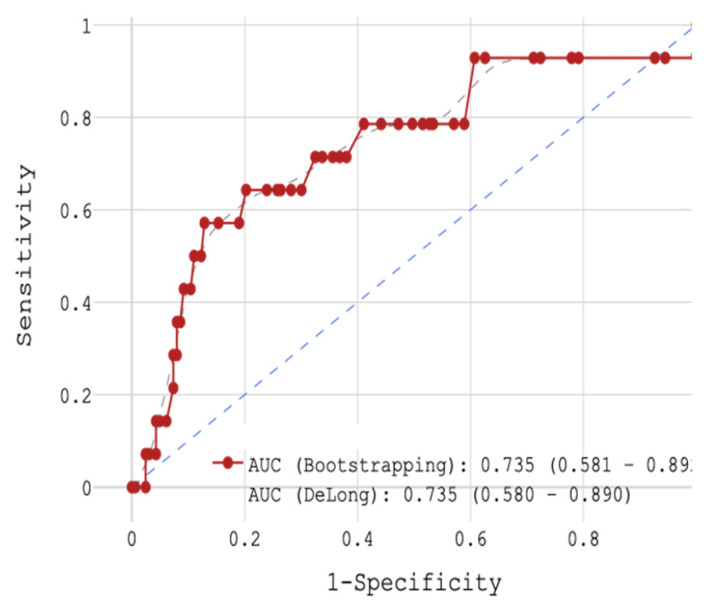
ROC-analysis by sST2 ≥ 12 ng/mL in-hospital mortality.

Patients were investigated with respect to in-hospital mortality depending on lower and higher sST2 threshold levels. With the use of ROC analysis, the whole group was divided into two groups: (1) cut-off < 12 ng/mL (n = 50) and (2) ≥ cut-off point 12 ng/mL (n = 127). The sensitivity was 64.3%, specificity 79.8%, and the accuracy 78.5% (AUC 0.735, Figure 6). In the 1st group, mortality was 2% (n = 1), and in the 2nd group 10.24% (n = 13).

The comparison between lower and higher sST2 is presented in Table 7 and Table 8. Here a difference in the ratio of men to women was observed (*p* = 0.007). In the ST2 ≥ 12 ng/mL group, there were higher concentrations of H-FABP, NT-proBNP, myoglobin, cystatin-C (*p* < 0.05).

**Figure 7:**
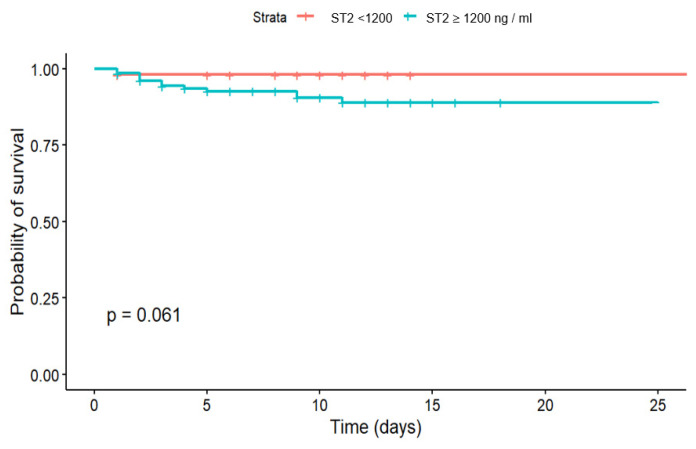
Kaplan-Meier hospital survival curves below and under sST2 cut-off 12 ng/mL.

The analysis based on Kaplan-Meier multiplier estimates and the Gehan-Wilcoxon test showed that there was a difference in-hospital survival for patients with sST2 > and ≤ 12 ng/mL with *p* = 0.061 (Figure 7).

In univariate analysis, three variables had high predictive value: glucose (*p* = 0.004) level, decrease of GRF (*p* = 0.003) and CRP > 11 U/mL (*p* = 0.009). CKD and DM variables were excluded from analysis to avoid doubling. The predictive model was effective with high LR = 29.36 (*p* < 0.001).

To identify significant predictors of in-hospital death among serum biomarkers, the Cox proportional hazards models were constructed. In such models, the biomarker, showed significant differences were considered to be independent covariates (Table 2), and glucose level and glomerular filtration rate were considered as controlled variables. As a result, when continuous variables (biomarkers levels) were included in the Cox models, no significant effect on in-hospital death was revealed: for LDL-C–HR = 0.339 (CI: 0.119–1.102, *p* = 0.073), Apo A1–HR = 0.998 (CI: 0.971–1.007, *p* = 0.209), NT-proBNP–HR = 1.0 (CI: 0.999–1.001, *p* = 0.542), Cystatin C–HR = 1.049 (CI: 0.282–3.900, *p* = 0.943), CRP–HR = 1.104 (CI: 0.826–1.477, *p* = 0.505), sST2–HR = 1.009 (CI: 0.996–1.024, *p* = 0.160), and LDH–HR = 1.001 (CI: 0.999–1.002, *p* = 0.229). However, after binarization of biomarker levels according to the cut-off points selected by ROC analysis, a model was found (Table 9) with a significant effect of CRP > 11 U/mL, for which HR = 4.928, CI 95%: 1.473–16.483 (*p* < 0.01). Of note, in all models, glucose levels and GFR were significant predictors of in-hospital mortality at a significance level of *p* < 0.01.

In addition to the obtained model, in which the binarized variable CRP > 11 mg/L was utilized with Cox proportional hazards regression as a predictor, univariate models with the inclusion of biomarker levels as continuous variables were also estimated. To ensure that the nonlinearity of the levels of CRP, H-FABP, and sST2 biomarkers was taken into account, Cox models with restricted cubic splines were estimated, following their pre-transformation due to the presence of skew in the distribution. Since H-FABP and sST2 had a right-sided skew, logarithmic transformation was applied, and for CRP, which has a left-sided skew, the values were “flipped” before logarithmic transformation by subtracting them from the maximum value with an added unit. The number of nodes for the splines was selected based on the minimum of the Akaike information criterion. Table 10 presents the results of estimating the Cox model with restricted cubic splines, with transformed values of biomarker levels taken into account as the only predictors (here HR and CI, 95% are given for transformed values).

**Figure 8:**
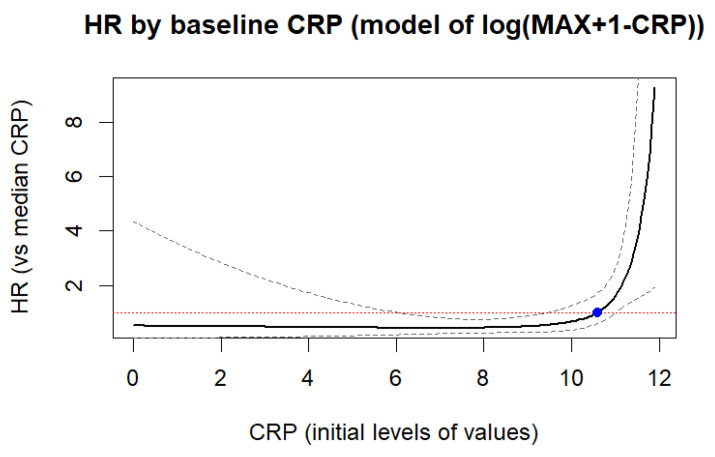
HR of hospital death was calculated on the basis of the Cox model using RCS (restricted cubic splines) based on CRP values. CRP optimal cut-off value was 10.65 mg/L aligning with the ROC-cut-off point 11.0 mg/L. The red line indicates the HR = 1 level (i.e., no risk), and the blue dotted line—the confidence interval range (CI) with 95% HR reliability.

As can be seen from the results, only the CRP level had a significant effect on hospital mortality, but in Table 10 the result is given for the transformed variable HR = 0.2855 (Figure 8). A graph of the dependence of the HR of hospital death on the original levels of CRP values was constructed for the original CRP variable, after the inverse transformation HR = 2.533, to ensure better understanding of the result obtained from assessment of the original values of CRP levels, and not from the transformed ones. Figure 8 shows HR of Hospital Death using Cox Models and RCS.

### 3.2. Survival and Mortality During Midterm FU

165 patients surviving hospital discharge were followed for 18 months (Table 11). Eighteen (10.1%) of 165 patients experienced CV death during FU. See Table 1 for patient characteristics by survival status. Additionally, CV events were captured during FU: 1 PE, 12 MI, 11 Stroke, and 69 CV hospitalizations were recorded in 1 year FU. FU deceased/survival differed by age (greater numbers of older patients were deceased, *p* = 0.045). DM and CKD were common comorbidities found in deceased FU patients (*p* < 0.05). Increased PA pressure (*p* = 0.017) and decreased LV EF (*p* = 0.026) was also observed in deceased FU patients. Results of biochemical and ELISA tests are presented in Table 12 and the groups differed only in sST2 concentration (*p* = 0.041, higher in deceased patients).

It must be reported that 6 patients experienced non-CV death and 3 patients were lost to follow-up in the post-discharge cohort, and were thus censored from CV-mortality analysis, see the Limitations section.

#### 3.2.1. CRP and Mortality During Midterm FU

**Figure 9:**
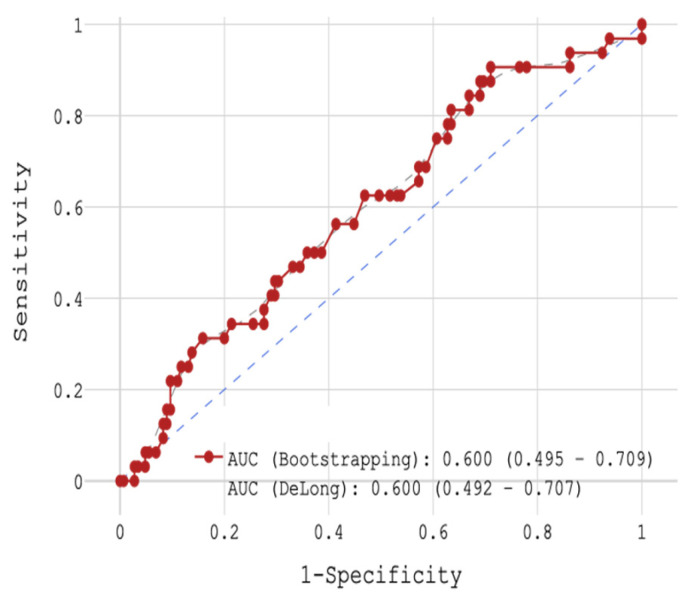
ROC-analysis by CRP in post-MI 2-years FU in STEMI patients.

In a next step, patients were investigated according to the occurrence of CV deaths with respect to CRP concentration during 18-month FU. With the help of ROC analysis, the group was divided into 2 groups: (1) with a cut-off < 8.1 mg/L (n = 45) and (2) with a cut-off ≥ 8.1 mg/L (n = 118). The sensitivity was 90.6%, specificity 29% and accuracy 40.1% (AUC 0.600, Figure 9). In the 1st group CV mortality was 6.52% (n = 3), and 22.14% (n = 29) in the 2nd group.

The groups were then compared with each other, see Table 13 and Table 14. The group with CRP ≥ 8.1 mg/L had lower LV EF (*p* = 0.008), lower cholesterol, Apo A1, Apo B1 (*p* < 0.05), but higher NT-proBNP and Cystatin-C (*p* < 0.01).

**Figure 10:**
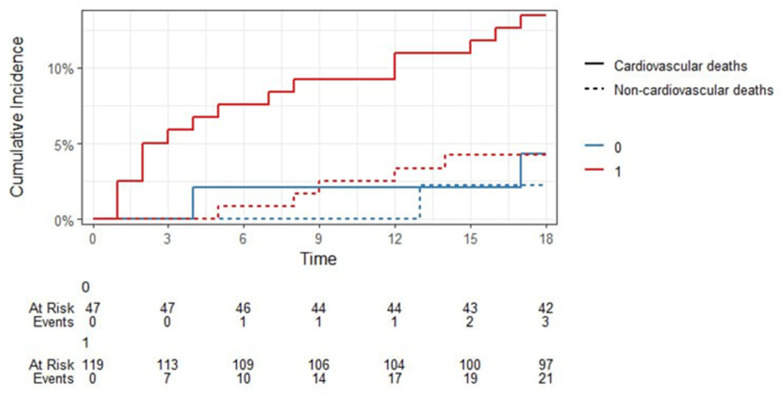
Fine-Gray estimates of the cumulative probability of survival up to 18 months after STEMI due to competing causes with CRP > 8.1 mg/L cut-off.

Since we had competing events (“Alive”, “Died from cardiovascular causes”, “Died from other causes”), we calculated their cumulative probability (Fine-Gray estimate) taking into account the competing risks of death after STEMI in the period up to 18 months for the CRP factor < 8.1 mg/L and ≥ 8.1 mg/L (binarized as 0 and 1, respectively, Figure 10). The results of the Fine-Gray test confirmed the significance of the effect of the CRP level exceeding 8.1 mg/L on mortality from cardiovascular causes at the level of *p* = 0.087, while for the event “Died from other causes” there was no significance of the effect (*p* = 0.53).

#### 3.2.2. H-FABP and Mortality During 18-Month Midterm FU

**Figure 11:**
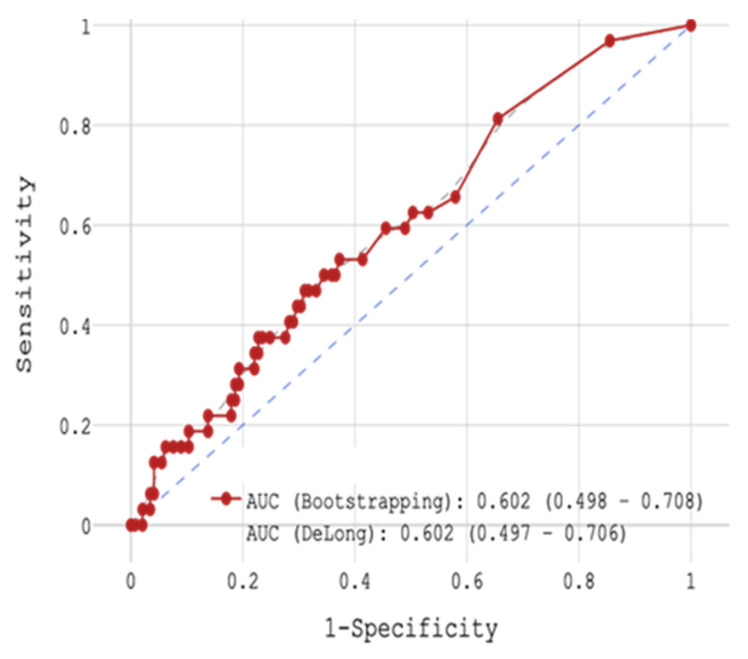
ROC-analysis by H-FABP in post-STEMI FU.

During 18-month FU, CV mortality with respect to H-FABP concentration was examined. With the use of ROC-analysis, the whole cohort was divided into 2 groups: (1) < 0.2 ng/mL cut-off (n = 57) and (2) ≥ 0.2 ng/mL (n = 106) cut-off with sensitivity 81.2%, specificity 34.5%, and accuracy 42.9% (AUC 0.602, Figure 11). In the 1st group, CV mortality during 18-month FU was 8.77% (n = 5), and in the 2nd group 12.64% CV mortality during FU (n = 13).

Table 15 compares the clinical and demographical characteristics of survived/deceased STEMI patients according to in H-FABP < 0.2 and ≥ 0.2 ng/mL cohorts. The H-FABP ≥ 0.2 ng/mL group showed higher SBP, prior stroke rate, and PA and also higher values in most of the cardio-specific biomarkers including CK-MB, NT-proBNP, TnI, myoglobin, cystatin-C and sST2 (*p* < 0.05) (Table 16).

For competing events (“Alive”, “Died from cardiovascular causes”, “Died from other causes”), the cumulative probability (Fine-Gray estimate) taking into account competing risks for H-FABP < ng/mL (binarized as 0 and 1, respectively) depending on the survival period in months is presented in Figure 11. The results of the Fine-Gray test did not confirm the significance of the effect of exceeding the H-FABP cut off point > 0.2 ng/mL on 18-months FU survival after STEMI (*p* = 0.45), for the event “Died from other causes” there was also no significance of the effect (*p* = 0.34).

#### 3.2.3. sST2 and Mortality During 18-Month Midterm FU

**Figure 12:**
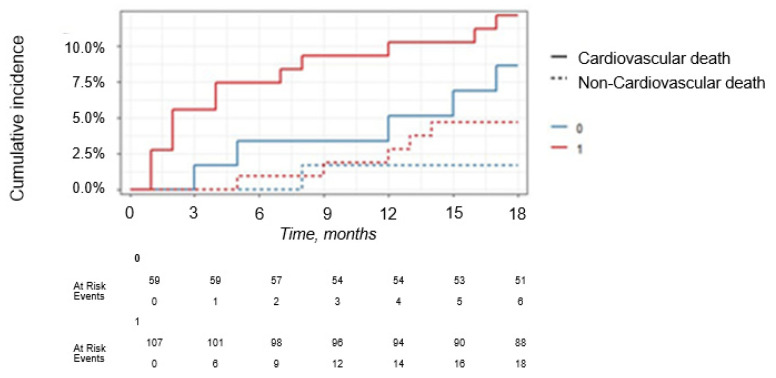
Fine-Gray estimates of the of cumulative survival probability after STEMI up to 18 months due to competing causes with H-FABP cut-off point > 0.2 ng/mL.

**Figure 13:**
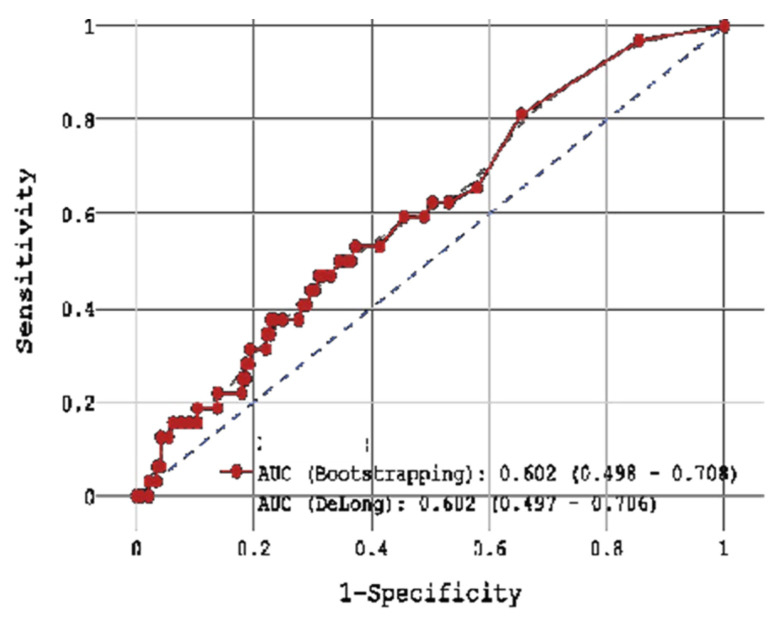
ROC-analysis at 18-months FU post discharge in STEMI patients by sST2.

Finally, patients were investigated during 18-month FU regarding CV mortality with respect to sST2 concentration. With the help of ROC-analysis (see Figure 12), the cohort was divided into 2 groups: (1) < 11 ng/mL cut-off (n = 88) and (2) ≥ 11 ng/mL cut-off (n = 75) with sensitivity 53.1%, specificity 62.8% and accuracy 61.4% (AUC 0.602, Figure 13). The mortality in the 1st group was 6.8% (n = 6), and in the 2nd group 6% (n = 12).

In Table 17 and Table 18, the comparison between the low/high sST2 values are presented. The group above the threshold differed in triglycerides, NT-proBNP, myoglobin, and sST2 only (*p* < 0.01, more in deceased patients).

**Figure 14:**
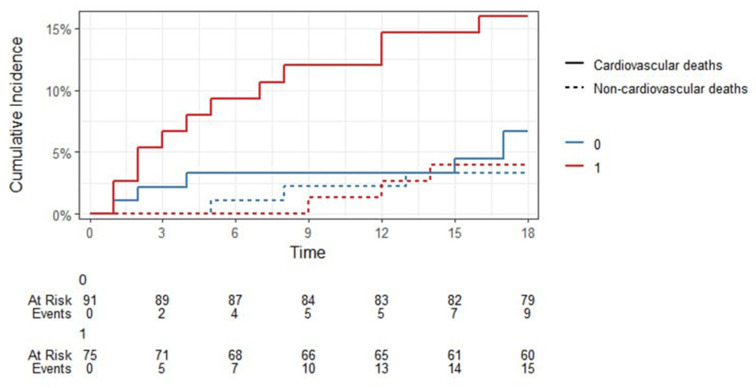
Fine-Gray estimates of cumulative survival probability after STEMI up to 18 months due to competing causes with sST2 cut-off > 11.0 ng/mL.

The cumulative probability (Fine-Gray estimate) taking into account the competing risks of the events “Alive”, “Died from cardiovascular causes”, “Died from other causes” for sST2 cut-off > 11 ng/mL (binarized as 0 and 1, respectively) for 18-month follow-up is shown in Figure 14. The results of the Fine-Gray test confirmed the significance of the effect of exceeding sST2 cut-off > 11.0 ng/mL on 18-month FU survival after STEMI (*p* = 0.05), for the event “Died from other causes” there was also no significance of the effect (*p* = 0.84).

**Figure 15:**
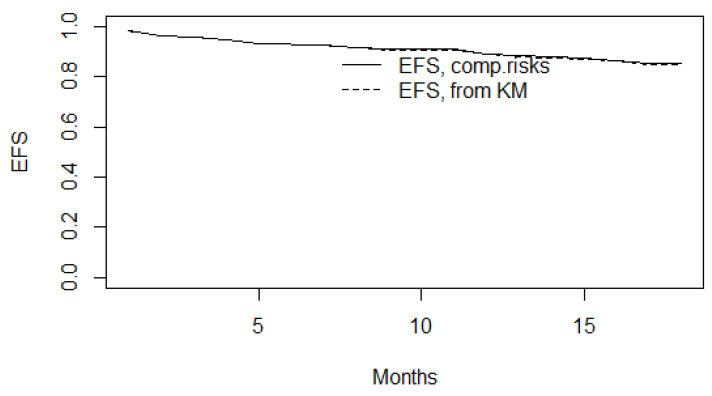
Event-free 18-month survival (EFS) estimated by CIF and Kaplan-Meier (KM) methods.

Since we had competing events (Alive, Cardiovascular Deaths, non-Cardiovascular Deaths) in our 18-month follow-up of patients after STEMI, in addition to 3 censored observations, event-free survival estimates were made. Figure 15 shows the 18-month event-free survival estimated by both the Cumulative Incidence Function (CIF) method and the Kaplan-Meier method.

To assess the risk of cardiovascular deaths, two-factor Fine-Gray models with competing risks (the event of interest is cardiovascular death) were constructed, where the biomarker levels (initial, transformed and binarized) and the patient’s age were considered as risk factors. For the right-skewed sST2 and H-FABP, logarithms were taken as a transformation, for the left-skewed CRP, the values were “inverted” from higher to lower before logarithmic transformation. The statistically significant model turned out to be the model taking into account the initial level of sST2 and the patient’s age, the results of which are summarized in Table 19.

To identify biomarkers as risk-factors of CV death in 18-month FU, Fine-Gray regressions were estimated with control variables of age. As a result, it was observed that sST2 ≥ 11 ng/mL and age had a significant effect at *p* < 0.05 on CV death during the 18-month FU period: HR = 1.01, CI 95% 1.00–1.02 and HR = 1.09, CI 95% 1.02–1.17, consequently.

## 4. Discussion

STEMI still represents a leading cause for CV morbidity and mortality worldwide and is thus also a considerable economic factor [1]. Although STEMI patients exhibit high in-hospital mortality, they also face a significant risk of major adverse CV events and CV death in the post-acute phase [14]. Identifying high-risk individuals after STEMI is therefore a key clinical objective. Despite this clear need, effective tools for risk stratification and prognosis remain limited, prompting ongoing research. Notably, many studies advocate for a multi-marker strategy, suggesting that combining biomarkers from diverse pathophysiological pathways may enhance diagnostic accuracy [15,16].

In this study, we aimed to assess the prognostic value of three CV biomarkers, CRP, H-FABP, and sST2, for mortality risk stratification in STEMI patients, both during hospitalization and over an 18-month FU period. Notably, these biomarkers represent distinct pathophysiological pathways, yet each has been associated with prognostic relevance in MI and related conditions, such as HF.

In our trial, 14 of the 179 patients died during baseline hospitalization for STEMI (7.8%) while the mortality rate in FU was much higher. Among the remaining 165 patients, 18 (10.9%) individuals died due to the CV reasons during the 18-month FU, while 6 died from non-CV causes.

Of the three biomarkers examined in our trial, only CRP > 11 mg/L together with low GFR (HR = 4.928, *p* = 0.004) and high glucose level (HR = 1.119, *p* = 0.004) showed potential for the prediction of in-hospital mortality in STEMI patients (Figure 2 and Figure 5 and Table 4 and Table 9), yet CRP was not robustly associated with CV mortality during 18-month FU. Regarding the cumulative probability of mortality during FU, the results of the Fine-Gray test confirmed the significance of the effect of the CRP level exceeding 8.1 mg/L on mortality from cardiovascular causes during FU at the level of *p* = 0.087, while for the event “Died from other causes” there was no significance of the effect (*p* = 0.53).

CRP is a downstream marker of systemic inflammation, participating in atherogenesis and atherothrombosis. CRP promotes endothelial dysfunction and accelerates the formation and progression of atherosclerotic plaques. In addition, it stimulates the production of proinflammatory cytokines, including interleukin 6 (IL-6) and tumor necrosis factor alpha (TNF-α), enhances oxidative stress, and contributes to a prothrombotic state. At sites of vascular injury or inflammation, CRP induces platelet activation and aggregation, directly contributing to thrombus formation and plaque instability, which are critical in ACS and sudden CV death [17]. Aligning with our findings, some prior studies have shown a correlation of increased CRP with in-hospital CV death. The Acuity multi-center US trial of 2974 patients with ACS linked higher baseline CRP levels to significantly higher incidence of CV mortality in patients with upper quartile baseline CRP values compared to those with the lowest quartile CRP values at 30 days post discharge (2.3 vs. 0.3%, *p* = 0.0004) and also at 1-year post discharge (5.5 vs. 2.8%, *p* = 0.0003) [18]. Several meta-analyses have also confirmed the prognostic value of CRP in acute coronary syndromes. He et al. performed a meta-analyses of longitudinal studies and showed that higher CRP within 72 h of the onset of ACS was associated with a moderate increased long-term risk of recurrent CV events or death, especially among patients with CRP exceeding 10 mg/L with pooled RR of 2.18, (95% CI: 1.77–2.68) for adverse outcomes compared to patients with low CRP ≤ 3 mg/L [19] One meta-analysis of seven heterogenous studies (six retrospective, one prospective) with more than 6000 STEMI patients observed that high pre-procedural (percutaneous coronary intervention) CRP values were associated with increased in-hospital all-cause mortality and CV events as well as increased post discharge all-cause mortality and CV events during 6-month FU. Due to the heterogeneity of the studies, however, CV mortality could not be ascertained [20], thus our study offers insight into CV mortality during hospitalization for STEMI.

In our study, association of elevated CRP with CV mortality in midterm FU was less robust contrasting with some studies found in the literature. A 2010 meta-analysis of 54 long-term studies of 160,309 patients without history of vascular disease demonstrated the association of CRP concentrations with the risk of later developing CHD, ischemic stroke and vascular mortality [21]. A recent analysis published in 2022 of high-risk vascular patients showed that higher longitudinal high-sensitivity CRP (hsCRP) levels over 30 months were independently associated with increased risk of major adverse CV events and all-cause death even in patients on optimal primary and secondary preventive therapies [22]. A cohort study performed in 2023 in patients with established CV disease with median 9.5-year FU found that higher CRP was independently associated with recurrent CV events and all-cause mortality, incidence also increasing with higher CRP levels and persisting in long-term FU to 15 years [21].

Although not measured in our study and a potential limitation, the use and predictive utility of the biomarker hsCRP must be mentioned. hsCRP is an independent predictor of short- and long-term CV events and CV mortality, yet its incremental predictive value compared to risk models using established risk factors is modest (i.e., risk models incorporating age, blood pressure, LDL-C, smoking status) with benefit shown when used to reclassify intermediate risk patients. In addition, hsCRP can be elevated in a range of non-CV conditions (i.e., infection, trauma, inflammatory diseases) limiting its specificity for CV risk assessment [23]. Standard CRP and hsCRP highly correlate in the acute phase at time of MI and demonstrate comparable diagnostic accuracy for predicting in-hospital mortality and major CV events following MI. Furthermore, several studies have demonstrated that elevated CRP measured within the first 24 h post-MI is independently associated with increased risk of long-term mortality and HF [24,25,26].

The second biomarker evaluated in our study for its potential association with in-hospital CV mortality and post-discharge CV mortality was H-FABP. In our study, the effectivity of H-FABP in prediction of in-hospital CV mortality risk was not confirmed, while in midterm FU differences in survival according to cut-off could be observed. The results of the Fine-Gray test did not confirm the significance of the effect of exceeding the H-FABP cut-off > 0.2 ng/mL on 18-months FU survival after STEMI (*p* = 0.45), and there was also no significance of the effect for the event “Died from other causes” (*p* = 0.34).

In the literature in contrast, H-FABP has been shown to have predictive value for CV events and CV mortality in several disease entities. Compared to established biomarkers, H-FABP provides complementary prognostic information to high-sensitivity cardiac troponin and NT-proBNP. H-FABP is more specific for myocardial injury and less confounded by non-cardiac inflammation than CRP. [27,28] While NT-proBNP and troponin remain superior for long-term risk stratification, H-FABP is particularly useful for early risk assessment. Contrasting with our results, elevated H-FABP levels measured within 12–24 h after ACS symptom onset in the literature are associated with a significantly increased risk of all-cause and CV mortality (4.1-fold higher HR), recurrent MI (HR 1.6), and developing HF (HR 4.5) over 10-months, independent of established risk factors and cardiac troponins, findings in contrast with our results [27,29,30]. In ACS, the prognostic value of H-FABP in troponin-negative patients has been described, identifying high-risk individuals missed by troponin alone [31]. In stable CAD, higher H-FABP also identifies individuals at higher risk for CV events and CV mortality, predicting a twofold increase in composite cardiovascular events and mortality over 24 months, independent of other clinical variables [32].

The predictive utility of H-FABP is varied and among patients with CKD, H-FABP is associated with major adverse CV events and increased non-CV mortality, although its association with CV mortality is less robust [28]. H-FABP has also been shown to be useful for the prediction of post-operative mortality and ventricular dysfunction following coronary artery bypass grafting [33]. In addition, H-FABP has been shown to be an independent predictor of all-cause as well as CV mortality in patients with chronic HF and DM type 2, with the strongest predictive utility among those with diabetes [34]. In our study, the effectivity of H-FABP in prediction of CV mortality risk was less robust, which we speculate may have been due to the relatively small sample size and/or the high rate of systemic thrombolysis.

One of the most important findings during the 18-month FU in our study was the predictive value of sST2 in predicting midterm CV mortality. sST2 > 11 ng/mL (HR = 5.101, *p* = 0.011) together with older age (HR = 1.022, *p* = 0.261), DM type 2 (HR = 6.433, *p* = 0.006) and decreased CKD (HR = 0.991, *p* = 0.991) showed a promising potential for the prediction of post-discharge mortality in 18-month FU among our STEMI patients (Figure 12 and Figure 13 and Table 16, Table 17 and Table 18). ST2 represents a marker of inflammation and cardiac stress and has two known isoforms: a membrane-bound ST2L and a soluble form, sST2 [35]. A ligand to both isoforms is interleukin-33 (IL-33), which is known to mediate cardioprotective effects on a molecular level through binding to the ST2L [36,37]. In contrast, sST2 acts as a decoy receptor, binding IL-33 and making it unavailable for cardioprotective signaling through the ST2L. This biomarker is elevated in numerous CV pathologies, such as HF, but also in MI [35].

sST2 was specifically analyzed in the multicenter, prospective PRIDE study (ProBNP Investigation of Dyspnea in the Emergency department) evaluating the diagnostic and prognostic utility of NT-proBNP in patients presenting with acute dyspnea. The study demonstrated that sST2 is a strong, independent predictor of mortality and heart failure hospitalization in patients with acute dyspnea, including those with acute heart failure and acute coronary syndrome [38]. In the context of ischemic heart disease, subsequent analyses of PRIDE showed that elevated sST2 levels at presentation were associated with increased risk of adverse outcomes, including all-cause mortality and incident heart failure, independent of traditional risk factors and established cardiac biomarkers. sST2 provided additive prognostic information to NT-proBNP and troponins, supporting its role in risk stratification for patients with acute coronary syndromes and heart failure [38] The additive prognostic value of sST2 to NT-proBNP and hs-TnT may reflect the pathophysiology underlying each biomarker. In contrast to NT-pBNP and troponin produced by cardiomyocytes, the production of sST2 finds its source in endothelial cells, and is promoted by tissue damage, inflammation, and extracellular matrix remodeling [39]. In PRIDE, patients with hemodynamic overload, expression of cardiomyocyte damage, and tissue remodeling showed an 8-fold increased risk of all-cause mortality and a 5-fold increased risk of CV mortality [39].

Several meta-analyses have demonstrated the predictive potential of sST2 not just in acute HF but in patients suffering from acute coronary syndromes [6,40]. A meta-analysis by Liu et comprised of 22 articles and 17,432 enrolled CAD patients showed that higher concentrations of baseline sST2 are associated with a higher risk of MACEs, all-cause mortality, CV death, and HF in patients with CAD. Elevated sST2 levels significantly predicted future MACEs in the ACS yet not in the non-ACS population [41]. However, sST2 in isolation cannot be considered as a risk factor. Its low specificity in relation to endpoints during MI was confirmed in the CLARITY-TIMI study [29,42]. However, as indicated by a sub-analysis of this trial, when sST2 is combined with NT-proBNP, the predictive prognostic power of short-term risk stratification in this population is enhanced. Nevertheless, the prognostic power of sST2 during a longer observation period has not yet been fully elucidated.

Furthermore, in our trial sST2 levels during baseline hospitalization were associated with less predictive accuracy when matched with the other two evaluated biomarkers aligning with the findings of the CLARITY-TIMI trial [37], although sST2 has shown efficacy in diagnosis of later CV events, CHF and even CV events after COVID-19 in FU [43]. Indeed, these findings support previous speculations, proposing a combination of cardiac biomarkers from different pathogenic backgrounds for improvement of risk stratification in different CV pathologies [11,21,25,38].

While our study focused on a STEMI population, some authors have noted comparable 1-year outcomes for STEMI and NSTEMI patients alike [44]. sST2 has also been shown to be an independent predictor of adverse outcomes in long-term follow-up after NSTEMI, demonstrating its predictive utility, especially when combined with traditional risk scores (e.g., GRACE). However, its value in improving outcomes over established risk models in a NSTEMI population is negligible [45]. In our study, the CV mortality rate of 22.1% during 18-month FU seems high. However, when compared to previous registry results, it is only slightly higher than CV endpoint rates in the average European population [39]. Our high CV mortality rate may be mainly attributed to our patient population stemming from large, rural regions with inadequate access to timely acute medical care and appropriate FU, also demonstrated by the high number of patients undergoing primary thrombolytic therapy (35/147, 23.8%) in our study. Additionally, high alcohol consumption, an unhealthy diet, a high incidence of metabolic syndrome and social-economic factors must be taken into account in this regard [40,41]. Nevertheless, high-risk patients can be effectively identified retrospectively, and our data emphasize the potential need for application of multimarker approaches in populations at increased risk for CV events.

Compared to previous trials, the cut-off levels for biomarkers proposed in our study are relatively divergent. Indeed, sST2 was shown to be a prognostic marker in the FU of HF patients, with a cut-off level of >35 ng/mL indicating a worse prognosis [21,23]. However, the calculated cut-off for sST2 for our study endpoint CV mortality was 27 ng/mL. The potential reasons for this finding are varied. First, differing results between labs may be attributed to the type of ELISA kits for sST2 analysis available, potentially accounting for variation in the sST2 values. Second, given the inclusion of patients up to 48 h after onset of symptoms, a delay in blood sampling may be a potential confounder in this regard, with sST2 levels peaking about 6–18 h after the onset of symptoms in MI [22,35]. Additionally, the young mean age of our study collective may have had an influence on the relatively low cut-off. Nevertheless, while dealing with CV mortality in a STEMI population with higher LVEF, considering the proposed cut-off value of 35 ng/mL in the FU of stable HF with reduced ejection fraction patients in numerous studies, our cut-off value seems reasonable [21,23]. On the other hand, regarding NT-pro-BNP, given the time between blood sampling and onset of symptoms as well as the ongoing secretion of NT-pro-BNP following MI, these findings also seem adequate.

In conclusion, our study proposes a significant correlation of biomarkers with 18-month post-discharge CV mortality in STEMI patients. While CRP > 11 mg/L has demonstrated prognostic efficacy predicting in-hospital mortality, our study could show that sST2 > 11 ng/mL is a promising predictive tool for CV mortality during 18-month FU post STEMI. In addition to CRP and sST2, the risk factors associated with in-hospital mortality were high glucose and low GFR, while older age and CKD were associated with higher mortality in midterm FU, which were in line with previous studies.

### 4.1. Future Perspectives

Currently, the American College of Cardiology/American Heart Association and European Society of Cardiology do not endorse use of CRP, H-FABP, or sST2 for risk stratification or prediction in acute coronary syndrome. However, our study and the aforementioned trials suggest the potential value of CRP and sST2 for targeted short- and intermediate CV event prediction, which in turn could guide the intensity of follow-up and therapeutic interventions.

CRP reflects residual inflammatory risk, and tailored post-discharge management of patients with higher CRP levels could thus include more intensive follow-up, aggressive risk factor modification, and consideration of anti-inflammatory therapies or statin intensification. sST2 reflects myocardial stress and fibrosis, and elevated sST2 levels are associated with a higher risk of heart failure, adverse remodeling, and mortality. As sST2 is not confounded by age or renal function, it may be valuable for use in elderly and comorbid populations.

Combining biomarkers with clinical risk scores (e.g., GRACE, TIMI), may enable more precise risk stratification, allowing clinicians to tailor follow-up intensity and guide therapeutic decisions for secondary prevention in the individual ischemic heart disease patient. Future, large prospective studies are needed to better determine the combined prognostic value of these biomarkers among specific STEMI subgroups by age, sex, ethnicity, and comorbidity profiles.

### 4.2. Limitations

Several limitations in our study must be considered. Our single-center study contained a relatively small sample size of n = 179. In this regard, the obtained sample of participants cannot be considered sufficiently representative, limiting the interpretation of results and its applicability to the general population. Furthermore, 9/156 patients (5.8%) in the post-discharge cohort were censored in Kaplan-Meier analysis: 6 due to non-CV death and 3 due to lost to follow-up. The survival status of the 3 lost to follow-up patients may only be speculated, although no information was found in the death registry.

A major limitation in our study was the lack of serialized biomarker measurements and our study used a single sample only which may not capture biomarker peaks at later timepoints. In our study, biomarkers were drawn within the first 8 h of admission prior to PCI, however, as CRP levels may peak later, the inflammatory signal in our study, therefore may be underrepresented. As previously mentioned, patients were included up to 48 h after onset of symptoms, therefore, a delay in blood sampling may be a potential confounder. While CRP was analyzed, our study also lacked hs-CRP analysis.

Another limitation of our study concerned the timing of the study blood sampling with respect to PCI or lytic therapies. In our study, there were high rates of thrombolytic therapy (35/147 patients, 23.8%), which is explained by longer patient transfer times from distant rural regions to our cardiac catheterization center. However, more specific information on the timing of the blood draw in the subgroup of patients undergoing lytic therapy was not analyzed, which is also a weakness of our paper. However, our data represent a real-life scenario, which in daily clinical practice is often characterized by testing and treatments done at various time points after symptom start/after presentation for STEMI. While hs-Troponin I and CK-MB were used to confirm the diagnosis of STEMI, hs-Tropinin T levels were not routinely used to facilitate the diagnosis of acute MI which may be considered a limitation. In addition, fast and routine applications of measurements of sST2 are currently still lacking. Therefore, despite promising results, routine application of the proposed biomarkers H-FABP and sST2 is still limited. It must be stated that due to our small sample size, our results are not intended to replace established risk scores (e.g., GRACE) but instead offer further insight into the prognostic potential of these biomarkers in STEMI patients.

## 5. Conclusions

Our findings highlight the clinical relevance of CRP and sST2 as prognostic biomarkers for CV mortality following STEMI. Elevated CRP levels on admission were independently associated with increased in-hospital mortality, while higher sST2 concentrations were linked to midterm post-discharge CV death. These associations underscore the potential of integrating both inflammatory and myocardial stress markers into existing risk stratification models. Although H-FABP showed less robust predictive value, its role within a multimarker approach merits further exploration. Taken together, the multimodal use of CRP and sST2, reflecting distinct pathophysiological pathways, may offer a more nuanced and practical strategy for identifying high-risk patients early and tailoring post-MI management. Future studies with larger multicenter cohorts are warranted to validate these biomarkers and refine their clinical application.

## Figures and Tables

**Table 1:** Clinical and demographical characteristics of STEMI patients survived/deceased in the hospital.

Parameter	All	Survivals	Deceased	*p*-Value
n	179	165	14	
Men, n (%)	124 (69.3)	116 (70.3)	8 (57.1)	0.306
Women, n (%)	55 (30.7)	49 (29.7)	6 (42.9)	0.306
Age, years	63.0 (53.5, 70.0)	63.0 (53.0, 70.0)	62.5 (57.5, 77.8)	0.340
Height, cm	170.0 (166.0, 174.5)	170.0 (166.0, 174.0)	172.5 (170.0, 178.8)	0.087
Weight, kg	78 (71.5, 87.5)	82 (72.0, 89.0)	81 (72.3, 88.3)	0.496
BMI, kg/m^2^	26.7 (24.1, 30.3)	26.6 (24.2, 30.3)	27.0 (22.4, 28.4)	0.615
Pulse, beat/min	74.0 (65.0, 85.0)	74.0 (66.0, 85.0)	75.0 (60.0, 83.0)	0.759
SBP, mm Hg	130.0 (115.0, 140.0)	130.0 (120.0, 140.0)	110.0 (90.0, 120.0)	0.001 **
DBP, mm Hg	80.0 (70.0, 90.0)	80.0 (70.0, 90.0)	70.0 (60.0, 75.0)	0.001 **
DM, n (%)	38 (21.0, 23.0)	30 (18.0, 18.0)	8 (57.0, 14.0)	0.001 ***
CKD, n (%)	29 (16.2)	23 (13.9)	6 (42.9)	0.005 **
AH, n (%)	175 (97.8)	162 (98.2)	13 (92.9)	0.725 ^‡^
Prior Stroke, n (%)	19 (10.6)	17 (10.3)	2 (14.3)	0.643
Dyslipidemia, n (%)	179 (100.0)	165 (100.0)	14 (100.0)	1.000
AF, n (%)	23 (12.9)	20 (12.1)	3 (21.4)	0.318
Prior MI, n (%)	35 (19.6)	32 (19.4)	3 (21.4)	0.854
Coronary angiography and revascularization				
Thrombolysis, n (%)	10 (5.6)	8 (4.9)	2 (14.30)	0.140
CAG, n (%)	175 (97.8)	161 (97.9)	14 (100.00)	0.140
LCA, n (%)	122 (68.2)	114 (69.1)	8 (57.1)	0.861
RCA, n (%)	65 (36.3)	60 (36.4)	5 (35.7)	0.962
LCx, n (%)	18 (10.1)	16 (9.7)	2 (14.30)	0.584
Stenting, n (%)	117 (65.4)	111 (67.3)	6 (42.9)	0.026 *
Echocardiography				
LV EF, %	54.0 (49.0, 60.0)	55.0 (49.8, 60.0)	46.5 (45.0, 56.8)	0.077
LV FS, %	28.0 (25.0, 32.0)	28.0 (25.0, 32.0)	24.0 (22.0, 29.5)	0.085
EDV, mm	4.8 (4.6, 5.1)	4.9 (4.6, 5.1)	4.7 (4.5, 4.8)	0.576
ESV, mm	3.5 (3.2, 3.8)	3.5 (3.2, 3.8)	3.1 (3.0, 3.4)	0.344
IVS, mm	1.2 (1.0, 1.2)	1.2 (1.0, 1.2)	1.1 (1.00, 1.2)	0.253
PA pressure, mm	34.5 (27.8, 45.0)	34.0 (27.0, 44.75)	44.5 (33.3, 55.3)	0.022 *

Values are either displayed as n-count with percent of the entire group in brackets or as median (quartile 1, quartile 3) for measured data. * *p* < 0.05, ** *p* < 0.01, *** *p* < 0.001. ^‡^—Yates correction was performed in the chi-square test. BMI: body mass index, SBP: systolic blood pressure, DBP: diastolic blood pressure, DM—Diabetes mellitus, CKD: chronic kidney disease, AH: arterial hypertension, AF: atrial fibrillation, MI: myocardial infarction, CAG: coronary angiography, LCA: left coronary artery, RCA: right coronary artery, LCx: left coronary artery, LV EF: left ventricle ejection fraction, LV ES: left ventricle ejection shortening, EDV: end diastolic volume, ESV: end systolic volume, IVS: interventricular septum, PA: pulmonary artery.

**Table 2:** Biochemical and ELISA parameters of STEMI patients survived/deceased in the hospital.

Parameter	All	Survivals	Deceased	*p*-Value
n	179	165	14	
CK-MB, U/L	24.3 (11.0; 70.0)	22.3 (10.8; 66.5)	49.5 (21.6, 72.1)	0.171
Cholesterol, mmol/L	4.6 (3.8, 5.6)	4.7 (3.8, 5.7)	4.15 (3.5, 4.6)	0.173
HDL-C, mmol/L	1.0 (0.8, 1.1)	1.0 (0.8, 1.1)	0.9 (0.8, 1.1)	0.281
Triglycerides, mmol/L	1.2 (0.9, 1.7)	1.2 (0.8, 1.6)	1.4 (1.0, 1.8)	0.194
LDL-C, mmol/L	1.9 (1.5, 2.4)	2.0 (1.5, 2.5)	1.7 (1.0, 1.9)	0.045 *
Glucose, mmol/L	6.5 (5.5, 8.6)	6.5 (5.4, 8.1)	8.9 (7.2, 20.2)	0.001 **
Magnesium, mmol/L	0.8 (0.7, 0.9)	0.8 (0.7, 0.9)	0.9 (0.7, 1.0)	0.133
Apo A1, g/L	128 (114.0, 151.0)	133.0 (114.5, 154.0)	117.0 (95.5, 122.3)	0.035 *
Apo B, g/L	107.0 (84.0, 133.0)	108.0 (85.0, 135.0)	100.5 (78.5, 125.3)	0.323
NT-proBNP, pg/mL	220.0 (20.0, 998.9)	210.3 (19.5, 776.3)	1523.4 (1104.1, 2537.0)	0.002 **
Tn I, ng/mL	4.4 (0.4, 14.7)	4.3 (0.4, 14.5)	6.0 (1.0, 15.9)	0.732
Myoglobin, ng/mL	39.2 (13.4, 145.5)	38.8 (12.3, 141.4)	104.9 (27.3, 223.0)	0.282
Cystatin C, mg/mL	1.0 (0.9, 1.2)	1.0 (0.8, 1.2)	1.2 (1.0, 1.5)	0.023 *
CRP, mg/L	10.6 (8.0, 11.0)	10.5 (7. 7, 10.9)	11.2 (10.7, 11.3)	0.004 **
H-FABP, ng/mL	0.6 (0.1, 4.3)	0.5 (0.1, 4.1)	1.9 (0.4, 6.6)	0.165
sST2, ng/mL	10.4 (0.0, 19.3)	10.2 (0.0, 17.3)	21.0 (13.7, 42.0)	0.004 **
Creatinine, mmol/L	87.5 (76.3, 106.0)	87 (76.0, 104.3)	115.5 (87.8, 134.0)	0.005 **
GFR, mL/min/m^2^	60.0 (48.0, 73.5)	61.0 (50.0, 74.0)	44.0 (32.5, 57.0)	0.003 **
Urea, mmol/L	6.0 (4.7; 8.0)	5.9 (4.7, 7.8)	11.0 (5.6, 20.5)	0.251
ALT, mmol/L	38.5 (27.0, 56.0)	37.6 (27.0, 55.3)	45.5 (26.5, 56.0)	0.393
AST, mmol/L	66.0 (41.3, 94.8)	62.5 (39.8, 94.0)	69.0 (49.0, 159.0)	0.277
LDH, mmol/L	605.0 (519.5, 961.0)	599.0 (502.0, 921.0)	985.0 (578.8, 1345.8)	0.046 *

Values are either displayed as n-count with percent of the entire group in brackets or as median (quartile 1, quartile 3) for measured data. * *p* < 0.05, ** *p* < 0.01. CK-MB: creatine kinase MB fraction, HDL-C: high density lipoprotein cholesterol, LDL-C: low density lipoprotein cholesterol, Apo A1: apolipoprotein A1, Apo B1: apolipoprotein B1, NT-pro BNP: N-terminal pro-B-type natriuretic peptide, Tn I: troponin I, CRP: C-reactive protein, H-FABP: heart-type fatty acid binding protein, ST2: suppression of tumorigenicity 2 protein, ALT: alanine aminotransferase, AST: aspartate aminotransferase, GFR: glomerular filtration rate, LDH: lactate dehydrogenase.

**Table 3:** Clinical and demographical characteristics of STEMI patients survived/deceased in hospital by CRP cut-off.

Parameter	CRP < 11 mg/L	CRP ≥ 11 mg/L	*p*-Value
n	135	42	
Men, n (%)	91 (67.4)	31 (73.8)	0.434
Women, n (%)	44 (32.6)	11 (26.2)	0.434
Age, years	62.0 (53.0, 70.0)	66.0 (60.0, 71.5)	0.066
Height, cm	170.0 (166.0, 175.0)	170.0 (166.5, 172.0)	0.967
Weight, kg	74.0 (71.0, 86.0)	82.0 (72.0 89.0)	0.224
BMI, kg/m^2^	26.3 (23.8, 29.3)	27.1 (25.2, 30.8)	0.275
Pulse, beat/min	74.0 (65.5, 85.5)	72.0 (65.0 79.5)	0.428
SBP, mm Hg	130.0 (112.5; 140.0)	130.0 (120.0, 140.0)	0.788
DBP, mm Hg	80.0 (70.0, 90.0)	80.0 (70.0, 90.0)	0.641
DM, n (%)	24 (17.8)	13 (30.1)	0.067
CKD, n (%)	19 (14.1)	10 (23.8)	0.137
HTN, n (%)	131 (97.0)	42 (100.0)	0.594 ^‡^
Prior Stroke, n (%)	15 (11.1)	4 (9.5)	0.772
Dyslipidemia, n (%)	135 (100.0)	42 (100.0)	1.000
AF, n (%)	19 (14.1)	4 (9.5)	0.444
Prior MI, n (%)	26 (19.2)	8 (19.0)	0.976
CAG and revascularization			
Thrombolysis, n (%)	4.0 (3.0)	6.0 (14.3)	0.006 **
CAG	132.0 (97.8)	41.0 (97.6)	0.594 ^‡^
LCA, n (%)	98.0 (72.6)	23.0 (54.8)	0.031 *
RCA, n (%)	46 (34.1)	19 (45.2)	0.190
LCx, n (%)	13 (9.6)	4 (9.5)	0.984
Stenting, n (%)	87 (64.4)	28 (66.7)	0.793
Echocardiographic parameters			
LV EF, %	54.0 (49.0, 60.0)	54.0 (50.0, 58.0)	0.876
LV FS, %	28.0 (25.0, 32.0)	28 (25.25.0, 31.5.0)	0.604
EDV, mm	4.8 (4.6, 5.1)	5.0 (4.7, 5.2)	0.934
ESV, mm	3.5 (3.2, 3.9)	3.3 (3.1, 3.6)	0.543
IVS, mm	1.1 (1.0, 1.2)	1.2 (1.0, 1.3)	0.827
PA pressure, mm	34.0 (27.5, 43.5)	37.0 (27.0, 49.0)	0.953

Values are either displayed as n-count with percent in brackets or as median (quartile 1, quartile 3) for measured data. * *p* < 0.05, ** *p* < 0.01, ^‡^—Yates correction was performed in the chi-square test; BMI: body mass index, SBP: systolic blood pressure, DBP: diastolic blood pressure, DM: diabetes mellitus, CKD: chronic kidney disease, AH: arterial hypertension, AF: atrial fibrillation, MI: myocardial infarction, CAG: coronary angiography, LCA: left coronary artery, RCA: right coronary artery, LCx: left coronary artery, LV EF: left ventricle ejection fraction, LV ES: left ventricle ejection shortening, EDV: end diastolic volume, ESV: end systolic volume, IVS: Interventricular Septum, PA: pulmonary artery.

**Table 4:** Biochemical analysis of STEMI survived/deceased patients in the hospital according to CRP cut-off.

Parameter	CRP < 11 mg/L	CRP ≥ 11 mg/L	*p*-Value
n	135	42	
CK-MB, U/l	24.3 (11.7, 70.1)	21.5 (8.0, 57.1)	0.549
Chol, mmol/L	4.8 (3.8, 5.7)	4.4 (3.8, 5.2)	0.191
HDL-C, mmol/L	1.0 (0.9, 1.2)	1.0 (0.8, 1.1)	0.145
Triglycerides, mmol/L	1.1 (0.75, 1.55)	1.5 (1.2, 2.0)	<0.001 ***
LDL-C, mmol	1.9 (1.4, 2.5)	1.9 (1. 5, 2.2)	0.449
Glucose, mmol/L	6.5 (5.4, 8.1)	7.5 (5.7, 11.0)	0.074
Magnesium, mmol/L	0.8 (0.7, 0.9)	0.83 (0.8, 0.9)	0.649
Apo A1, g/L	134.0 (116.0, 157.5)	118.5 (106.0, 133.0)	0.003 **
Apo B, g/L	111.0 (85.5, 135.5)	103.5 (80.8, 125.8)	0.308
NT-proBNP, pg/mL	215.0 (12.1, 792.9)	264.5 (47.0, 1477.0)	0.065
TnI, ng/mL	4.6 (0.6, 15.0)	3.9 (0.3, 11.5)	0.862
Myoglobin, ng/mL	42.1 (15.6, 184.4)	26.4 (9.7, 117.5)	0.184
Cystatin C, mg/mL	1.0 (0.8, 1.2)	1.0 (0.9, 1.2)	0.076
H-FABP, ng/mL	0.6 (0.1, 6.9)	0.3 (0.1, 2.0)	0.240
sST2, ng/mL	12.2 (0.0, 17.9)	6.8 (0.0; 23.2)	0.657
Creatinine, mmol/L	87 (76.0, 103.3)	88.5 (78.0, 117.8)	0.250
GFR, mL/min/m^2^	60.0 (50.5, 74.0)	58.0 (43.0, 69.5)	0.176
Urea, mmol/L	5.7 (4.6, 7.6)	76.0 (4.9, 13.0)	0.122
ALT, mmol/L	39.0 (28.0, 56.0)	37.6 (24.0; 55.0)	0.923
AST, mmol/L	66.0 (39.25, 94.0)	62.5 (44.5, 96.8)	0.611
LDH, mmol/L	593.0 (521.0, 880.5)	820.0 (528.0, 1026.8)	0.218

Values are either displayed as n-count with percent in brackets or as median (quartile 1, quartile 3) for measured data. ** *p* < 0.01, *** *p* < 0.001. CK-MB: creatine kinase MB fraction, HDL-C: high density lipoprotein cholesterol, LDL-C: low density lipoprotein cholesterol, Apo A1: apolipoprotein A1, Apo B1: apolipoprotein B1, NT-proBNP: N-terminal pro-B-type natriuretic peptide, Tn I: troponin I, CRP: C-reactive protein, H-FABP: heart-type fatty acid binding protein, sST2: soluble suppression of tumorigenicity 2 protein, ALT: alanine aminotransferase, AST: aspartate aminotransferase, GFR: glomerular filtration rate, LDH: lactate dehydrogenase.

**Table 5:** Clinical and demographical characteristics of STEMI patients survived/deceased in hospital by H-FABP cut-off </≥ 0.7 ng/mL.

Parameter	H-FABP < 0.7 ng/mL	H-FABP ≥ 0.7 ng/mL	*p*-Value
n	92	85	
Men, n (%)	64 (69.6)	58 (68.24)	*p* = 0.849
Women, n (%)	28 (30.4)	27 (31.76)	*p* = 0.849
Age, years	63.5 (53.0, 70.0)	62.0 (56.0, 70.0)	*p* = 0.988
Height, cm	170.0 (166.8, 174.0)	170.0 (166.0, 176.0)	*p* = 0.968
Weight, kg	78.0 (72.0, 86.3)	76 (70.0, 88.0)	*p* = 0.836
BMI, kg/m^2^	26.6 (24.1, 30.3)	26.5 (24.2, 30.3)	*p* = 0.945
Pulse, beat/min	74.0 (67.5, 86.5)	72.0 (65.0, 80.0)	*p* = 0.172
SBP, mm Hg	130.0 (120.0, 150.0)	130.0 (110.0, 140.0)	*p* = 0.006 **
DBP, mm Hg	80.0 (70.0 90)	80.0 (70.0, 85.0)	*p* = 0.021 *
DM, n (%)	17 (18.5)	20 (23.5)	*p* = 0.409
CKD, n (%)	10 (10.9)	19 (22.4)	*p* = 0.040 *
AH, n (%)	91 (98.9)	82 (96.5)	*p* = 0.558 ^‡^
Prior Stroke, n (%)	6 (6.5)	13 (15.3)	*p* = 0.060
Dyslipidemia, n (%)	92 (100.0)	85 (100.0)	*p* = 1.000
AF, n (%)	12 (13.0)	11 (12.9)	*p* = 0.984
Prior MI, n (%)	14 (15.2)	20 (23.5)	*p* = 0.837
CAG and revascularization			
Thrombolysis, n (%)	3 (3.3)	7 (8.2)	*p* = 0.153
CAG, n (%)	90 (97.8)	83 (97.7)	*p* = 0.937
LCA, n (%)	62 (67.4)	59 (69.4)	*p* = 0.773
RCA, n (%)	38 (41.3)	27 (31.8)	*p* = 0.189
LCx, n (%)	8 (8.7)	9 (10.6)	*p* = 0.670
Stenting, n (%)	62 (67.4)	53 (62.4)	*p* = 0.483
Echocardiographic data			
LV EF, %	54.0 (49.8, 60.3)	54.0 (47.8, 58.5)	*p* = 0.282
LV FS, %	28.0 (25.0, 32.0)	28.0 (24.0, 32.0)	*p* = 0.349
EDV, mm	4.9 (4.7, 5.1)	4.7 (4.6, 5.1)	*p* = 0.359
ESV, mm	3.4 (3.2, 3.9)	3.6 (3.2, 3.8)	*p* = 0.724
IVS, mm	1.1 (1.0, 1.2)	1.2 (1.0, 1.2)	*p* = 0.774
PA pressure, mm	33.0 (27.0; 41.0)	36.5 (28.8, 47.3)	*p* = 0.064

Values are either displayed as n-count with percent in brackets or as median (quartile 1, quartile 3) for measured data. * *p* < 0.05, ** *p* < 0.01. ^‡^—Yates correction was performed in the chi-square test; BMI: body mass index, SBP: systolic blood pressure, DBP: diastolic blood pressure, DM: Diabetes mellitus, CKD: chronic kidney disease, AH: arterial hypertension, AF: atrial fibrillation, MI: myocardial infarction, CAG: coronary angiography, LCA: left coronary artery, RCA: right coronary artery, LCx: left coronary artery, LV EF: left ventricle ejection fraction, LV ES: left ventricle ejection shortening, EDV: end diastolic volume, ESV: end systolic volume, IVS: interventricular septum, PA: pulmonary artery.

**Table 6:** Biochemical analysis of STEMI survived/deceased patients in the hospital according to H-FABP cut-off.

Parameter	H-FABP < 0.7 ng/mL	H-FABP ≥ 0.7 ng/mL	*p*-Value
n	92	85	
CK-MB, U/L	13.5 (6.9, 22.6)	66.5 (27.8, 122.2)	0.001 ***
Chol, mmol/L	4.5 (3.8, 5.6)	4.8 (3.8, 5.7)	0.707
HDL-C, mmol/L	1.0 (0.8, 1.1)	1.0 (0.9, 1.2)	0.209
Trigylcerides, mmol/L	1.4 (1.03, 1.87)	1.0 (0.8, 1.5)	0.002 **
LDL-C, mmol/L	1.9 (1.5, 2.4)	1.9 (1.2, 2.4)	0.459
Glucose, mmol/L	6.1 (5.1, 8.4)	7.0 (5.8, 8.9)	0.031 *
Magnesium, mmol/L	0.8 (0.7, 0.9)	0.8 (0.7, 0.9)	0.759
Apo A1, g/L	124.0 (113.8, 144.0)	133.0 (114.0, 161.0)	0.327
Apo B, g/L	111.0 (89.0, 131.3)	107.0 (81.0, 137.0)	0.610
NT-proBNP, pg/mL	164.2 (19.7, 748.5)	370.0 (30.0, 1359.7)	0.099
TnI, ng/mL	1.8 (0.1, 7.0)	11.6 (2.6, 18.3)	0.001 ***
Myoglobin, ng/mL	14.0 (8.7, 29.7)	143.4 (62.0, 587.4)	0.001 ***
Cystatin C, mg/mL	1.0 (0.8, 1.1)	1.0 (0.9, 1.2)	0.058
CRP, mg/L	10.7 (7.7, 11.0)	10.0 (8.3, 10.9)	0.399
sST2, ng/mL	8.2 (0.0, 14.9)	11.7 (0.0, 21.0)	0.001 ***
Creatinine, mmol/L	87.0 (71.0, 101.0)	89.0 (78.0, 106.0)	0.209
GFR, mL/min/m^2^	62.5 (48.0, 76.0)	59.0 (50.0, 68.0)	0.215
Urea, mmol/L	5.3 (4.7, 7.7)	6.0 (4.8, 8.2)	0.752
ALT, mmol/L	35.0 (23.0, 53.3)	43.5 (28.0, 56.0)	0.148
AST, mmol/L	56.0 (37.0, 89.0)	68.5 (44.9, 114.0)	0.102
LDH, mmol/L	594.5 (511.8; 960.0)	615.0 (550.0, 967.0)	0.846

Values are either displayed as n-count with percent in brackets or as median (quartile 1, quartile 3) for measured data. * *p* < 0.05, ** *p* < 0.01, *** *p* < 0.001. CK-MB: creatine kinase MB fraction, HDL-C: high density lipoprotein cholesterol, LDL-C: low density lipoprotein cholesterol, Apo A1: apolipoprotein A1, Apo B1: apolipoprotein B1, NT-proBNP: N-terminal pro-B-type natriuretic peptide, Tn I: troponin I, CRP: C-reactive protein, H-FABP: heart-type fatty acid binding protein, sST2: soluble suppression of tumorigenicity 2 protein, ALT: alanine aminotransferase, AST: aspartate aminotransferase, GFR: glomerular filtration rate, LDH: lactate dehydrogenase.

**Table 7:** Clinical and demographical characteristics of in-hospital survived/deceased STEMI patients by sST2 cut-off.

Parameter	sST2 < 12 ng/mL	sST2 ≥ 12 ng/mL	*p*-Value
n	50	127	
Men, n (%)	42 (84.0)	80 (63.0)	0.007 **
Women, n (%)	8 (16.0)	47 (37.0)	0.007 **
Age, years	62.5 (53.5, 69.8)	63.0 (55.0, 70.0)	0.785
Height, cm	170.0 (168.0, 174.8)	170.0 (165.5, 174.5)	0.434
Weight, kg	74.0 (71.0, 86.0)	76.0 (70.0, 88.0)	0.369
BMI, kg/m^2^	27.4 (24.5, 30.2)	26.2 (23.9, 30.3)	0.312
Pulse, beat/min	72.0 (65.3, 84.3)	74.0 (65.0, 84.0)	0.637
SBP, mm Hg	140.0 (120.0, 150.0)	130.0 (110.0, 140.0)	0.025
DBP, mm Hg	80.0 (70.0, 90.0)	80.0 (70.0, 85.0)	0.231
DM, n (%)	9 (18.0)	28 (22.1)	0.552
CKD, n (%)	7 (14.0)	22 (17.3)	0.591
AH, n (%)	50 (100.0)	123 (96.9)	0.480 ^‡^
Prior Stroke, n (%)	4 (8.0)	15 (11.8)	0.461
Dyslipidemia, n (%)	50 (100.0)	127 (100.0)	1.000
AF, n (%)	6 (12.0)	17 (13.4)	0.806
Prior MI, n (%)	7 (14.0)	27 (21.3)	0.270
CAG and revascularization			
Thrombolysis, n (%)	3 (6.0)	7 (5.5)	0.123
CAG, n (%)	50 (100.0)	123 (96.9)	0.480 ^‡^
LCA, n (%)	37 (74.0)	84 (66.1)	0.312
RCA, n (%)	16 (32.0)	49 (38.6)	0.414
LCx, n (%)	3 (6.0)	14 (11.0)	0.324
Stenting, n (%)	35 (70.0)	80 (63.0)	0.379
Echocardiographic parameters			
LV EF, %	55.0 (50.0, 58.8)	54.0 (49.0, 60.8)	0.883
LV FS, %	28.0 (25, 31.0)	28.0 (25.0, 32.0)	0.800
EDV, mm	4.8 (4.6, 5.0)	4.9 (4.6, 5.1)	0.866
ESV, mm	3.5 (3.3, 4.0)	3.4 (3.1, 3.7)	0.194
IVS, mm	1.1 (1.0, 1.2)	1.2 (1.0, 1.2)	0.992
PA pressure, mm	33.0 (27.0 40.0)	35 (28.0, 45.0)	0.239

Values are either displayed as n-count with percent in brackets or as median (quartile 1, quartile 3) for measured data. ** *p* < 0.01. ^‡^—Yates correction was performed in the chi-square test; BMI: body mass index, SBP- systolic blood pressure, DBP: diastolic blood pressure, DM: Diabetes mellitus, CKD: chronic kidney disease, AH: arterial hypertension, AF: atrial fibrillation, MI: myocardial infarction, CAG: coronary angiography, LCA: left coronary artery, RCA: right coronary artery, LCx: left coronary artery, LV EF: left ventricle ejection fraction, LV ES: left ventricle ejection shortening, EDV: end diastolic volume, ESV: end systolic volume, IVS: interventricular septum, PA: pulmonary artery.

**Table 8:** Biochemical analysis of STEMI survived/deceased patients in hospital according to sST2 cut-off.

Parameter	sST2 < 12 ng/mL	sST2 ≥ 12 ng/mL	*p*-Value
n	50	127	
CK-MB, U/L	20.7 (10.8, 33.2)	27.8 (11.7, 73.7)	0.133
Chol, mmol/L	4.9 (4.0, 5.7)	4.5 (3.8, 5.6)	0.399
HDL-C, mmol/L	1.0 (0.8, 1.2)	1.0 (0.8, 1.1)	0.696
Triglycerides, mmol/L	1.3 (1.1, 1.7)	1.2 (0.8, 1.6)	0.179
LDL-C, mmol/L	2.1 (1.6, 2.5)	1.9 (1.4, 2.4)	0.144
Glucose, mmol/L	6.1 (5.1, 8.4)	6.6 (5.6, 8.7)	0.268
Magnesium, mmol/L	0.8 (0.8, 0.9)	0.8 (0.7, 0.9)	0.307
Apo A1, g/L	131.0 (115.5, 157.8)	127.0 (112.0 147.5)	0.395
Apo B, g/L	119.0 (93.0, 137.5)	107.0 (81.0, 131.0)	0.087
NT-pBNP, pg/mL	30.8 (3.1, 196.3)	370.0 (59.2, 1398.8)	<0.001 ***
TnT, ng/mL	2.6 (0.5, 8.3)	5.9 (0.4, 17.4)	*p* = 0.558
Myoglobin, ng/mL	22.6 (9.7, 63.1)	46.3 (18.2, 198.0)	*p* = 0.009 **
Cystatin C, mg/mL	1.0 (0.8, 1.1)	1.0 (0.9, 1.2)	*p* = 0.019 *
CRP, mg/L	10.5 (6.3, 11.0)	10.6 (8.9, 10.9)	*p* = 0.517
H-FABP, ng/mL	0.2 (0.1, 2.1)	0.8 (0.1, 6.4)	*p* = 0.007 **
Creatinine, mmol/L	96.0 (76.5, 108.8)	86.0 (76.3, 103.8)	*p* = 0.329
GFR, mL/min/m^2^	57.0 (47.3, 70.8)	61.0 (50.0, 74.0)	*p* = 0.397
Urea, mmol/L	6.4 (5.0, 7.7)	5.8 (4.3, 10.7)	*p* = 0.353
ALT, mmol/L	42 (30.8, 61.8)	36.0 (26.0, 55.0)	*p* = 0.114
AST, mmol/L	67.5 (44.0, 94.5)	64.5 (40.3, 94.0)	*p* = 0.431
LDH, mmol/L	574.0 (472.5, 828.5)	635.0 (538.5, 968.5)	*p* = 0.207

Values are either displayed as n-count with percent in brackets or as median (quartile 1, quartile 3) for measured data. * *p* < 0.05, ** *p* < 0.01, *** *p* < 0.001. CK-MB: creatine kinase MB fraction, HDL-C: high density lipoprotein cholesterol, LDL-C: low density lipoprotein cholesterol, Apo A1: apolipoprotein A1, Apo B1: apolipoprotein B1, NT-proBNP: N-terminal pro-B-type natriuretic peptide, Tn I: troponin I, CRP: C-reactive protein, H-FABP: heart-type fatty acid binding protein, sST2: soluble suppression of tumorigenicity 2 protein, ALT: alanine aminotransferase, AST: aspartate aminotransferase, GFR: glomerular filtration rate, LDH: lactate dehydrogenase.

**Table 9:** Cox regression analysis of binarized variables for hospital survivals.

Parameter	HR	CI, 95%	Harrell’s CI (SE), LR (*p*-Value)	*p*-Value
Glucose, mmol/L	1.119	1.037–1.207	0.863 (SE = 0.044) LR = 29.36 (*p* < 0.001)	0.004 **
GFR, mL/min/m^2^	0.960	0.934–0.987		0.003 **
CRP > 11 mg/L	4.928	1.473–16.483		0.009 **

Values are displayed as n median (quartile 1, quartile 3). ** *p* < 0.01.

**Table 10:** Univariant Cox regression analysis with Restricted Cubic Splines (RCS).

Parameter	Type of Transformation	Effect	S.E. of Effect	HR ^1^	CI, 95% ^1^
CRP, mg/L	Log (max(CRP) + 1-CRP)	−1.2537 **	0.4611	0.2855	0.116–0.705
H-FABP, ng/mL	log	1.6935	0.9335	5.4387	0.873–33.893
sST2, ng/mL	log	1.9392	1.0603	6.9533	0.870–55.549

^1^—calculated for transformed biomarker values; ** *p* < 0.01. Table 10: Univariant Cox regression analysis with restricted cubic splines using logarithmic transformation due to skewed biomarker values.

**Table 11:** Clinical and demographical characteristics of STEMI survived/deceased patients in post-discharge FU.

Parameter	Hospital Survivals	FU Survivals	FU Deceased	*p*-Value
n	165	147	18	
Men, n (%)	116 (70.3)	104 (70.8)	12 (66.7)	0.721
Women, n (%)	49 (29.7)	43 (29.3)	6 (33.3)	0.721
Age, years	63.0 (53.0, 70.0)	62.0 (53.0, 69.5)	68.0 (61.3, 76.5)	0.045 *
Height, cm	170.0 (166.0, 174.0)	170.0 (166.0, 174.0)	170.5 (166.5, 174.0)	0.709
Weight, kg	82.0 (72.0, 89.0)	74.0 (70.0, 84.0)	81.0 (70.3, 87.5)	0.538
BMI, kg/m^2^	26.6 (24.2, 30.3)	26.5 (24.2, 30.2)	27.3 (24.3, 30.4)	0.651
Pulse, beat/min	74.0 (66.0, 85.0)	74.0 (65.0, 85.0)	78.0 (70.5, 84.5)	0.342
SBP, mm Hg	130.0 (120.0, 140.0)	130.0 (120.0, 145.0)	120.0 (110.0, 140.0)	0.173
DBP, mm Hg	80.0 (70.0, 90.0)	80.0 (70.0, 90.0)	75.0 (70.0, 80.0)	0.139
DM, n (%)	30 (18.2)	23 (15.7)	7 (38.9)	0.016 *
CKD, n (%)	23 (14.0)	17 (11.6)	6 (33.3)	0.012 *
AH, n (%)	162 (98.2)	145 (98.6)	17 (94.4)	0.747 ^‡^
Prior Stroke, n (%)	17 (10.3)	13 (8.8)	4 (22.2)	0.079
Dyslipidemia, n (%)	165 (100.0)	147 (100.0)	18 (100.0)	1.000
AF, n (%)	20 (12.1)	18 (12.3)	2 (11.1)	0.890
Prior MI, n (%)	32 (19.4)	26 (17.7)	6 (33.3)	0.114
CAG and revascularization				
Thrombolysis, n (%)	10 (5.6)	8 (5.4)	0 (0.0)	0.665 ^‡^
CAG, n (%)	175 (97.8)	144 (98.0)	17 (94.4)	0.918 ^‡^
LCA, n (%)	122 (68.2)	102 (69.4)	12 (66.7)	0.814
RCA, n (%)	65 (36.3)	52 (35.4)	8 (44.4)	0.451
LCx, n (%)	18 (10.1)	14 (9.5)	2 (11.1)	0.860
Stenting, n (%)	117 (65.4)	101 (68.7)	10 (55.6)	0.109
Echocardiographic data				
LV EF, %	54.0 (49.0, 60.0)	55.0 (51.0, 60.0)	50.5 (45.5, 56.3)	0.026 *
LV FS, %	28.0 (25.0, 32.0)	28.0 (25.0, 32.0)	25.5 (23.0, 28.0)	0.020 *
EDV, mm	4.8 (4.6, 5.1)	4.8 (4.6, 5.2)	5.0 (4.7, 5.1)	0.036 *
ESV, mm	3.5 (3.2, 3.8)	3.5 (3.2, 3.9)	3.3 (3.3, 3.6)	0.280
IVS, mm	1.2 (1.0, 1.2)	1.2 (1.0, 1.2)	1.2 (1.1, 1.2)	0.326
PA pressure, mm	34.5 (27.8, 45.0)	33.0 (27.0, 42.0)	44.0 (31.0, 60.8)	0.017 *

Values are either displayed as n-count with percent in brackets or as median (quartile 1, quartile 3) for measured data. * *p* < 0.05. ^‡^—Yates correction was performed in the chi-square test; BMI: body mass index, SBP—systolic blood pressure, DBP: diastolic blood pressure, DM: diabetes mellitus, CKD: chronic kidney disease, AH: arterial hypertension, AF: atrial fibrillation, MI: myocardial infarction, CAG: coronary angiography, LCA: left coronary artery, RCA: right coronary artery, LCx: left coronary artery, LV EF: left ventricle ejection fraction, LV ES: left ventricle ejection shortening, EDV: end diastolic volume, ESV: end systolic volume, IVS: interventricular septum, PA: pulmonary artery.

**Table 12:** Biochemical and ELISA analysis of survived/deceased STEMI patients during post-discharge FU.

Parameter	Hospital Survivals	FU Survivals	FU CV Deaths	*p*-Value
n	165	147	18	
CK-MB, U/L	22.30 (10.80, 66.50)	23.40 (10.80, 67.90)	18.90 (11.25, 37.03)	*p* = 0.749
Chol, mmol/L	4.70 (3.83, 5.68)	4.70 (3.80, 5.70)	4.7 (3.90, 5.00)	*p* = 0.360
HDL-C, mmol/L	0.99 (0.83, 1.13)	1.0 (0.8, 1.1)	0.95 (0.84,1.06)	*p* = 0.353
Triglyceride, mmol/L	1.23 (0.84, 1.60)	1.23 (0.86, 1.59)	0.99 (0.75, 1.79)	*p* = 0.476
LDL-C, mmol/L	1.99 (1.47, 2.46)	2.01 (1.48, 2.47)	1.79 (1.39, 2.33)	*p* = 0.468
Glucose, mmol/L	6.46 (5.43, 8.12)	6.31 (5.28, 7.87)	8.11 (6.17, 8.94)	*p* = 0.014
Magnesium, mmol/L	0.80 (0.72, 0.88)	0.8 (0.74, 0.88)	0.72 (0.63, 0.86)	*p* = 0.117
Apo A1, g/L	133.00 (114.50, 154.00)	133.00 (115.00, 157.00)	124.50 (113.50, 140.30)	*p* = 0.471
Apo B1, g/L	108.00 (85.00, 135.00)	111.00 (87.00, 137.00)	101.50 (76.30, 116.75)	*p* = 0.087
NT-pBNP, pg/mL	210.32 (19.5.0, 776.3.0)	215.0 (18.95, 807.81)	171.41 (22.50, 562.10)	*p* = 0.853
TnT, ng/mL	4.30 (0.40, 14.50)	4.00 (0.40, 14.80)	5.65 (1.10, 11.30)	*p* = 0.523
Myoglobin, ng/mL	38.80 (12.30, 141.40)	36.50 (12.30, 126.90)	77.00 (13.88, 440.30)	*p* = 0.174
Cystatin C, mg/mL	1.00 (0.80, 1.20)	1.00 (0.80, 1.20)	1.00 (0.90, 1.20)	*p* = 0.493
CRP, mg/L	10.50 (7.67, 10.90)	10.50 (7.10, 10.91)	10.45 (9.38, 10.81)	*p* = 0.989
H-FABP, ng/mL	0.50 (0.10, 4.10)	0.50 (0.10, 3.70)	1.10 (0.13, 14.73)	*p* = 0.229
sST2, ng/mL	10.20 (0.00, 17.25)	9.00 (0.00, 16.90)	14.90 (9.45, 22.23)	*p* = 0.041 *
Creatinine, mmol/L	87.00 (76.00, 104.25)	87.00 (76.00, 101.75)	86.50 (79.00, 123.75)	*p* = 0.236
GFR, mL/min/m^2^	61.00 (50.00, 74.00)	61.00 (51.00, 74.00)	59.00 (34.75, 73.50)	*p* = 0.213
Urea, mmol/L	5.9 0(4.73, 7.75)	5.70 (4.70, 7.60)	12.10 (11.8, 14.60)	*p* = 0.263
ALT, mmol/L	37.55 (27.00, 55.25)	37.50 (27.00, 56.75)	38.55 (27.25, 48.00)	*p* = 0.583
AST, mmol/L	62.50 (39.75, 94.00)	67.00 (41.30, 97.30)	49.50 (34.25, 68.75)	*p* = 0.101
LDH, mmol/L	599.00 (502.00, 921.00)	599.00 (519.50, 910.00)	614.00 (467.30, 965.30)	*p* = 0.919

Values are either displayed as n-count with percent in brackets or as median (quartile 1, quartile 3) for measured data. * *p* < 0.05. CK-MB: creatine kinase MB fraction, HDL-C: high density lipoprotein cholesterol, LDL-C: low density lipoprotein cholesterol, Apo A1: apolipoprotein A1, Apo B1: apolipoprotein B1, NT-proBNP: N-terminal pro-B-type natriuretic peptide, Tn I: troponin I, CRP: C-reactive protein, H-FABP: heart-type fatty acid binding protein, sST2: soluble suppression of tumorigenicity 2 protein, ALT: alanine aminotransferase, AST: aspartate aminotransferase, GFR: glomerular filtration rate, LDH: lactate dehydrogenase.

**Table 13:** Clinical and demographical characteristics of STEMI patients survived/deceased during post-discharge FU by CRP cut-off < 8.1 mg/L.

Parameter	CRP < 8.1 mg/L	CRP ≥ 8.1 mg/L	*p*-Value
n	45	118	
Men, n (%)	32 (71.1)	82 (69.5)	*p* = 0.841
Women, n (%)	13 (28.9)	36 (30.5)	*p* = 0.841
Age, years	61.0 (52.0, 70.0)	64.0 (56.0, 70.0)	*p* = 0.105
Height, cm	170.0 (168.0, 174.0)	170.0 (165.0, 174.0)	*p* = 0.996
Weight, kg	74.0 (70.0, 84.0)	79.0 (72.0, 88.0)	*p* = 0.184
BMI, kg/m^2^	26.1 (23.7, 28.1)	26.8 (24.3, 30.5)	*p* = 0.159
Pulse, beat/min	72.0 (64.0, 86.0)	74.0 (66.0, 83.5)	*p* = 0.398
SBP, mm Hg	130.0 (110.0, 150.0)	130.0 (120.0, 140.0)	*p* = 0.406
DBP, mm Hg	80.0 (70, 90.0)	80.0 (70.0, 90.0)	*p* = 0.412
DM, n (%)	5 (11.1)	24 (20.3)	*p* = 0.169
CKD, n (%)	7 (15.6)	16 (13.6)	*p* = 0.744
AH, n (%)	45 (100.0)	115 (97.5)	*p* = 0.669 ^‡^
Prior Stroke, n (%)	4 (8.9)	13 (11.0)	*p* = 0.692
Dyslipidemia, n (%)	45 (100.0)	118 (100.0)	*p* = 1.000
AF, n (%)	5 (11.1)	15 (12.7)	*p* = 0.781
Prior MI, n (%)	5 (11.1)	26 (22.0)	*p* = 0.113
CAG and revascularization			
Thrombolysis, n (%)	2 (4.4)	6 (5.1)	*p* = 0.866
CAG, n (%)	44 (97.8)	115 (97.5)	*p* = 0.906 ^‡^
LCA, n (%)	35 (77.8)	78 (66.1)	*p* = 0.149
RCA, n (%)	12 (26.7)	48 (40.7)	*p* = 0.098
LCx, n (%)	3 (6.7)	12 (10.2)	*p* = 0.490
Stenting, n (%)	27 (60)	82 (69.5)	*p* = 0.250
Echocardiographic parameters			
LV EF, %	57. (52.0, 62.0)	53.0 (49.0, 58.0)	*p* = 0.008 **
LV FS, %	29.0 (25.0, 33.0)	27.0 (25.0, 31.0)	*p* = 0.058
EDV, mm	4.7 (4.6, 4.9)	4.9 (4.7, 5.1)	*p* = 0.296
ESV, mm	3.5 (3.2, 3.8)	3.5 (3.2, 3.8)	*p* = 0.306
IVS, mm	1.1 (1.0, 1.3)	1.2 (1.0, 1.2)	*p* = 0.398
PA pressure, mm	33.0 (27.0, 39.0)	34.0 (27.0, 45.0)	*p* = 0.391

Values are either displayed as n-count with percent in brackets or as median (quartile 1, quartile 3) for measured data. ** *p* < 0.01. ^‡^—Yates correction was performed in the chi-square test; BMI—body mass index, SBP—systolic blood pressure, DBP—diastolic blood pressure, DM- Diabetes mellitus, CKD—chronic kidney disease, AH—arterial hypertension, AF—atrial fibrillation, MI- myocardial infarction, CAG—coronary angiography, LCA—left coronary artery, RCA—right coronary artery, LCx—left coronary artery, LV EF—left ventricle ejection fraction, LV ES—left ventricle ejection shortening, EDV—end diastolic volume, ESV—end systolic volume, IVS- Interventricular Septum, PA- pulmonary artery.

**Table 14:** Biochemical and ELISA tests of STEMI survived/deceased patients in FU according to CRP cut-off value.

Parameter	CRP < 8.1 mg/L	CRP ≥ 8.1 mg/L	*p*-Value
n	45	118	
CK-MB, U/L	23.40 (9.30, 39.50)	20.70 (10.90, 68.53)	0.530
Chol, mmol/L	5.00 (4.50, 6.00)	4.50 (3.70, 5.30)	0.007 **
HDL-C, mmol/L	1.04 (0.87, 1.29)	0.97 (0.82, 1.10)	0.147
Triglyceride, mmol/L	1.05 (0.83, 1.52)	1.28 (0.90, 1.71)	0.132
LDL-C, mmol/L	2.21 (1.81, 2.59)	1.81 (1.37, 2.37)	0.025 *
Glucose, mmol/L	6.31 (5.10, 7.53)	6.50 (5.50, 8.37)	0.285
Magnesium, mmol/L	0.82 (0.73, 0.90)	0.79 (0.71, 0.87)	0.300
Apo A1, g/L	150.00 (118.00, 169.00)	124.50 (111.00, 144.00)	0.001 **
Apo B1, g/L	122.00 (106.00, 148.00)	103.00 (83.25, 130.00)	0.003 **
NT-proBNP, pg/mL	33.16 (0.00; 331.26)	286.15 (45.80, 1082.80)	<0.001 ***
TnI, ng/mL	3.70 (0.40, 12.50)	5.80 (0.45, 15.23)	0.679
Myoglobin, ng/mL	43.30 (14.90, 126.90)	38.05 (1148.00, 144.00)	0.867
Cystatin C, mg/mL	0.90 (0.70; 1.00)	1.00 (0.90; 1.20)	0.004 **
H-FABP, ng/mL	0.60 (0.10, 6.10)	0.45 (0.10, 3.20)	0.717
sST2, ng/mL	9.00 (0.00, 14.80)	12.20 (0.00, 20.50)	0.136
Creatinine, mmol/L	87.00 (76.00, 101.00)	86.00 (75.00, 103.00)	0.847
GFR, mL/min/m^2^	60.00 (53.00, 73.00)	61.00 (50.00, 74.00)	0.704
Urea, mmol/L	5.00 (4.80, 7.00)	6.00 (4.65, 9.55)	0.879
ALT, mmol/L	39.00 (31.00, 47.00)	37.00 (26.00, 56.00)	0.795
AST, mmol/L	57.00 (37.75, 89.00)	66.00 (44.00, 94.00)	0.319
LDH, mmol/L	579.00 (540.00, 8.00)	619.00 (497.30, 959.80)	0.574

Values are either displayed as n-count with percent in brackets or as median (quartile 1, quartile 3) for measured data. * *p* < 0.05, ** *p* < 0.01, *** *p* < 0.001. CK-MB: creatine kinase MB fraction, HDL-C: high density lipoprotein cholesterol, LDL-C: low density lipoprotein cholesterol, Apo A1: apolipoprotein A1, Apo B1: apolipoprotein B1, NT-proBNP: N-terminal pro-B-type natriuretic peptide, Tn I: troponin I, CRP: C-reactive protein, H-FABP: heart-type fatty acid binding protein, sST2: soluble suppression of tumorigenicity 2 protein, ALT: alanine aminotransferase, AST: aspartate aminotransferase, GFR: glomerular filtration rate, LDH: lactate dehydrogenase.

**Table 15:** Clinical and demographical characteristics of survived/deceased STEMI patients according to H-FABP cut-off in 18-month post-discharge FU.

Parameter	H-FABP < 0.2 ng/mL	H-FABP ≥ 0.2 ng/mL	*p*-Value
n	57	106	
Men, n (%)	44 (77.2)	77 (66.0)	*p* = 0.527
Women, n (%)	13 (22.8)	29 (34.0)	*p* = 0.527
Age, years	62.0 (52.0, 68.0)	65.0 (56.0, 70.0)	*p* = 0.138
Height, cm	170.0 (168.0, 174.0)	170.0 (164.3, 174.0)	*p* = 0.328
Weight, kg	80.0 (72.0, 86.0)	74.0 (71.0, 88.0)	*p* = 0.520
BMI, kg/m^2^	25.9 (24.1, 30,4)	26.8 (24.2, 30.0)	*p* = 0.818
Pulse, beat/min	74.0 (66.0, 90.0)	73.0 (65.3, 82.0)	*p* = 0.485
SBP, mm Hg	135.0 (125.0, 150.0)	130.0 (111.3,140.0)	*p* = 0.034 *
DBP, mm Hg	80.0 (70.0, 90.0)	80.0 (70.0, 86.3)	*p* = 0.221
DM, n (%)	11 (19.3)	18 (17.0)	*p* = 0.713
CKD, n (%)	3 (5.3)	20 (18.9)	*p* = 0.018 *
AH, n (%)	57 (100.0)	103 (97.2)	*p* = 0.503 ^‡^
Prior Stroke, n (%)	2 (3.5)	15 (14.2)	*p* = 0.035 *
Dyslipidemia, n (%)	57 (100.0)	106 (100.0)	*p* = 1.000
AF, n (%)	7 (12.3)	13 (12.3)	*p* = 0.988
Prior MI, n (%)	7 (12.3)	24 (22.6)	*p* = 0.108
CAG and stenting			
Thrombolysis, n (%)	1 (1.8)	7 (6.6)	*p* = 0.324 ^‡^
CAG	57 (100.0)	102 (96.0)	*p* = 0.341 ^‡^
LCA, n (%)	39 (68.4)	74 (69.8)	*p* = 0.855
RCA, n (%)	26 (45.6)	34 (32.1)	*p* = 0.088
LCx, n (%)	4 (7.0)	11 (10.4)	*p* = 0.480
Stenting, n (%)	41 (71.9)	68 (64.2)	*p* = 0.315
Echocardiographic data			
LV EF, %	55.0 (51.0, 60.0)	54.0 (48.0, 60.0)	*p* = 0.458
LV FS, %	28.0 (26.0, 32.0)	28.0 (25.0, 32.0)	*p* = 0.628
EDV, mm	4.9 (4.8, 5.1)	4.8 (4.6, 5.1)	*p* = 0.491
ESV, mm	3.5 (3.2, 3.9)	3.5 (3.2, 3.8)	*p* = 0.673
IVS, mm	1.1 (1.0, 1.2)	1.2 (1.0, 1.2)	*p* = 0.313
PA pressure, mm	320 (26.5, 39.5)	35.0 (28.0, 45.0)	*p* = 0.035 *

Values are either displayed as n-count with percent in brackets or as median (quartile 1, quartile 3) for measured data. * *p* < 0.05. ^‡^—Yates correction was performed in the chi-square test; BMI: body mass index, SBP: systolic blood pressure, DBP: diastolic blood pressure, DM: diabetes mellitus, CKD: chronic kidney disease, AH: arterial hypertension, AF: atrial fibrillation, MI: myocardial infarction, CAG: coronary angiography, LCA: left coronary artery, RCA: right coronary artery, LCx: left coronary artery, LV EF: left ventricle ejection fraction, LV ES: left ventricle ejection shortening, EDV: end diastolic volume, ESV: end systolic volume, IVS: interventricular septum, PA: pulmonary artery.

**Table 16:** Biochemical analysis of STEMI survived/deceased patients in FU according to H-FABP cut-off.

Parameter	H-FABP < 0.2 ng/mL	H-FABP ≥ 0.2 ng/mL	*p*-Value
n	57	106	
CK-MB, U/L	10.80 (6.30, 20.10)	38.85 (17.10, 108.70)	*p* < 0.001 ***
Chol, mmol/L	4.50 (3.90, 5.60)	4.80 (3.80, 5.70)	*p* = 0.652
HDL-C, mmol/L	0.96 (0.83, 1.10)	1.0 (0.84, 1.15)	*p* = 0.547
Triglycerides, mmol/L	1.4 (0.99, 1.86)	1.1 (0.80, 1.52)	*p* = 0.039
LDL-C, mmol/L	1.98 (1.55, 2.48)	1.97 (1.43, 2.43)	*p* = 0.515
Glucose, mmol/L	5.99 (5.10, 7.87)	6.68 (5.48, 8.42)	*p* = 0.136
Magnesium, mmol/L	0.80 (0.73, 0.88)	0.79 (0.72, 0.88)	*p* = 0.910
Apo A1, g/L	132.00 (116.00, 144.00)	133.50 (113.25, 160.25)	*p* = 0.737
Apo B, g/L	111.00 (89.00, 130.00)	107.50 (84.00, 135.75)	*p* = 0.707
NT-proBNP, pg/mL	66.32 (9.47, 595.85)	311.50 (30.79, 800.37)	*p* = 0.043 *
TnI, ng/mL	1.20 (0.10, 70)	7.00 (1.03, 17.95)	*p* = 0.004 **
Myoglobin, ng/mL	10.00 (8.3.0, 16.20)	71.60 (36.08, 278.63)	*p* < 0.001 ***
Cystatin C, mg/mL	0.90 (0.80, 1.00)	1.05 (0.90, 1.20)	*p* = 0.008 **
CRP, mg/L	10.63 (8.30, 11.00)	10.35 (7.10, 10.85)	*p* = 0.274
sST2, ng/mL	6.30 (0, 13.60)	13.45 (0.78, 22.23)	*p* = 0.006 **
Creatinine, mmol/L	87.00 (76.75, 98.00)	85.00 (76.25, 105.00)	*p* = 0.709
GFR, mL/min/m^2^	64.00 (50.00, 72.00)	60.00 (51.00, 75.50)	*p* = 0.581
Urea, mmol/L	6.05 (4.80, 7.60)	5.80 (4.40, 8.10)	*p* = 0.892
ALT, mmol/L	35.00 (26.00, 52.00)	40.00 (28.00, 56.00)	*p* = 0.567
AST, mmol/L	56.00 (36.00, 86.00)	68.00 (44.00, 108.00)	*p* = 0.131
LDH, mmol/L	584.00 (515, 902.00)	599.5 (507.50, 959.00)	*p* = 0.618

Values are either displayed as median (quartile 1, quartile 3) for measured data. * *p* < 0.05, ** *p* < 0.01, *** *p* < 0.001. CK-MB: creatine kinase MB fraction, HDL-C: high density lipoprotein cholesterol, LDL-C: low density lipoprotein cholesterol, Apo A1: apolipoprotein A1, Apo B1: apolipoprotein B1, NT-proBNP: N-terminal pro-B-type natriuretic peptide, Tn I: troponin I, CRP: C-reactive protein, H-FABP: heart-type fatty acid binding protein, sST2: soluble suppression of tumorigenicity 2 protein, ALT: alanine aminotransferase, AST: aspartate aminotransferase, GFR: glomerular filtration rate, LDH: lactate dehydrogenase.

**Table 17:** Clinical and demographical characteristics of survived/deceased STEMI patients according to ST2 cut-off at 18-month post-discharge FU.

Parameter	sST2 < 11 ng/mL	sST2 ≥ 11 ng/mL	*p*-Value
n	88	75	
Men, n (%)	66 (75.0)	48 (64.0)	0.127
Women, n (%)	22 (24.2)	27 (36.0)	0.127
Age, years	63.0 (54.8, 69.3)	64.0 (52.5, 70.0)	0.808
Height, cm	170.0 (166.0, 174.0)	170.0 (165.5, 174.5)	0.892
Weight, kg	82.0 (72.0, 89.0)	74.0 (70.0, 84.5)	0.108
BMI, kg/m^2^	27.7 (24.4, 31.0)	25.8 (23.9, 28.4)	0.070
Pulse, beat/min	72.0 (65.0, 82.5)	76.0 (67.0, 86.0)	0.233
SBP, mm Hg	135(120, 150)	130.0 (110.0, 140.0)	0.033
DBP, mm Hg	80 (70, 90)	80.0 (70.0, 85.0)	0.169
DM, n (%)	19 (21.6)	10 (13.3)	0.170
CKD, n (%)	11 (12.5)	12 (16.0)	0.523
AH, n (%)	87 (98.9)	73 (97.3)	0.889 ^‡^
Prior Stroke, n (%)	7 (8.0)	10 (13.3)	0.263
Dyslipidemia, n (%)	88 (100.0)	75 (100.0)	1.000
AF, n (%)	8 (9.1)	12 (16.0)	0.181
Prior MI, n (%)	16 (18.2)	15 (20.0)	0.769
CAG and revascularization			
Thrombolysis, n (%)	5 (5.7)	3 (4.0)	0.621
CAG, n (%)	85 (96.6)	74 (98.7)	0.730 ^‡^
LCA, n (%)	62 (70,5)	51 (68.0)	0.420
RCA, n (%)	30 (34.1)	30 (40.0)	0.436
LCx, n (%)	6 (6.8)	9 (12.0)	0.254
Stenting, n (%)	60 (68.2)	49 (65.3)	0.701
Echocardiographic data			
LV EF, %	55.0 (51.0, 59.5)	54.0 (48.5, 60.5)	0.702
LV FS, %	28.0 (25.0, 32.0)	28.0 (24.5, 32.0)	0.985
EDV, mm	4.8 (4.6, 5.1)	4.9 (4.7, 5.1)	0.240
ESV, mm	3.5 (3.2, 4.0)	3.5 (3.1, 3.7)	0.218
IVS, mm	1.2 (1.1, 1.3)	1.1 (1.0, 1.2)	0.158
PA pressure, mm	33.0 (27, 40.0)	35.0 (28.0, 45.0)	0.206

Values are either displayed as n-count with percent in brackets or as median (quartile 1, quartile 3) for measured data. ^‡^—Yates correction was performed in the chi-square test; BMI: body mass index, SBP: systolic blood pressure, DBP: diastolic blood pressure, DM: Diabetes mellitus, CKD: chronic kidney disease, AH: arterial hypertension, AF: atrial fibrillation, MI: myocardial infarction, CAG: coronary angiography, LCA: left coronary artery, RCA: right coronary artery, LCx: left coronary artery, LV EF: left ventricle ejection fraction, LV ES: left ventricle ejection shortening, EDV: end diastolic volume, ESV: end systolic volume, IVS: Interventricular Septum, PA: pulmonary artery.

**Table 18:** Biochemical analysis of survived/deceased STEMI patients in 18-months FU according to sST2 cut-off.

Parameter	sST2 < 11 ng/mL	sST2 ≥ 11 ng/mL	*p*-Value
n	88	75	
CK-MB, U/L	21.15 (9.68, 37.93)	26.10 (11.70, 107.80)	0.070
Chol, mmol/L	4.85 (4.08, 6.00)	4.50 (3.60, 5.60)	0.078
HDL-C, mmol/L	1.00 (0.85, 1.16)	0.96 (0.83, 1.1)	0.448
Triglycerides, mmol/L	1.30 (0.98, 1.84)	1,03 (0.73, 1.49)	0.004 **
LDL-C, mmol/L	2.04 (1.64, 2.53)	1.73 (1.20, 2.36)	0.071
Glucose, mmol/L	6.41 (5.21, 8.65)	6.50 (5.60, 7.93)	0.743
Magnesium, mmol/L	0.80 (0.75, 0.88)	0.80 (0.55, 0.88)	0.117
Apo A1, g/L	134.50 (117.00, 162.00)	123.00 (110.00, 143.50)	0.024
Apo B, g/L	115.50 (96.50, 138.3.00)	104.00 (78.50, 131.00)	0.013
NT-proBNP, pg/mL	63.95 (4.74, 366.90)	460.92 (146.84, 1367.50)	<0.001 ***
Tn, ng/mL	3.25 (0.38, 9.78)	7.00 (0.50, 18.10)	0.307
Myoglobin, ng/mL	29.35 (9.83, 76.13)	49.10 (19.60, 222.90)	0.009 **
Cystatin C, mg/mL	1.00 (0.88, 1.10)	1.10 (0.80, 1.25)	0.042 *
CRP, mg/L	10.48 (6.21, 11.02)	10.54 (9.35, 10.81)	0.890
H-FABP, ng/mL	0.20 (0.10, 2.70)	0.80 (0.20, 7.60)	0.009 **
Creatinine, mmol/L	88.50 (77.50, 102.75)	84.50 (75.0, 102.25)	0.421
GFR, mL/min/m^2^	60.00 (50.75, 71)	63.00 (50.50, 78.50)	0.422
Urea, mmol/L	6.40 (5.10, 8.00)	4.80 (4.00, 6.70)	0.062
ALT, mmol/L	39.00 (26.75, 55.50)	35.50 (28.00, 54.50)	0.652
AST, mmol/L	62.50 (42.00, 92.25)	62.00 (40.25, 105.50)	0.659
LDH, mmol/L	579.50 (526.25, 859.25)	621.00 (498.50, 938.50)	0.679

Values are either displayed as as median (quartile 1, quartile 3) for measured data. * *p* < 0.05, ** *p* < 0.01, *** *p* < 0.001. CK-MB: creatine kinase MB fraction, HDL-C: High Density Lipoprotein Cholesterol, LDL-C: Low Density Lipoprotein Cholesterol, Apo A1_ apolipoprotein A1, Apo B1: apolipoprotein B1, NT-proBNP: N-terminal pro-B-type natriuretic peptide, Tn I: troponin I, CRP: C-reactive protein, H-FABP: heart-type fatty acid binding protein, sST2: soluble suppression of tumorigenicity 2 protein, ALT: alanine aminotransferase, AST: aspartate aminotransferase, GFR: glomerular filtration rate, LDH: lactate dehydrogenase.

**Table 19:** Fine-Gray survival model evaluating CV mortality in 18-month FU.

Parameter	Coef (SE)	HR	CI, 95%	*p*
Age, years	0.085 (0.035)	1.09	1.02–1.17	0.016 *
sST2, ng/mL	0.013 (0.005)	1.01	1.00–1.02	0.017 *

* *p* < 0.05.

## Data Availability

Data is available upon request from the first and last authors k.kopp@salk.at and znaufal@mail.ru.

## Abbreviations

The following abbreviations are used in this manuscript:

ACS	acute coronary syndrome
AF	atrial fibrillation
AH	arterial hypertension
ALT	alanine aminotransferase
Apo A1	apolipoprotein A1
Apo B1	apolipoprotein B1
AST	aspartate aminotransferase
AUC	area under the curve
BMI	body mass index
CAD	coronary artery disease
CAG	coronary angiography
CHD	coronary heart disease
CI	confidence interval
CKD	chronic kidney disease
CK-MB	myoglobin fraction of creatine phosphokinase
cm	centimeters
COPD	chronic obstructive pulmonary disease
CRP	C-reactive protein
CV	cardiovascular
DBP	diastolic blood pressure
DM	diabetes mellitus
ECG	electrocardiogram
EDV	end diastolic volume
ELISA	enzyme immunoassay
ESC	European Society of Cardiology
ESV	end systolic volume
FU	follow-up
GCP	good clinical practice
GFR	glomerular filtration rate
h	hours
HDL-C	high-density lipoprotein cholesterol
HF	heart failure
H-FABP	hear-type fatty acid binding protein
HR	hazard ratio
HTN	hypertension
kg	kilograms
IL-1	interleukin-1
IL-6	interleukin-6
IL-33	interleukin-33
LR	likelihood ratio test
IVS	interventricular septum
LCA	left coronary artery
LDH	lactate dehydrogenase
LCx	left coronary artery
LDL-C	low-density lipoprotein cholesterol
LVEF	left ventricle ejection fraction
LVES	left ventricle ejection shortening
MI	myocardial infarction
m^2^	square meters
min	minutes
mL	milliliters
mm	millimeters
mm Hg	millimetre of mercury
ng	nanograms
NT-proBNP	N-terminal pro-B-type natriuretic peptide
PA	pulmonary artery
RCA	right coronary artery
PCI	percutaneous catheter intervention
SBP	systolic blood pressure
sST2	soluble suppression of tumorigenicity 2
STEMI	ST-segment elevation myocardial infarction
TNF-α	tumor necrosis factor alpha
Tn I	troponin I
U/L	units per liter
